# Therapeutic body wraps (TBW) for treatment of severe injurious behaviour in children with autism spectrum disorder (ASD): A 3-month randomized controlled feasibility study

**DOI:** 10.1371/journal.pone.0198726

**Published:** 2018-06-29

**Authors:** Pierre Delion, Julien Labreuche, Dominique Deplanque, David Cohen, Alain Duhamel, Céline Lallié, Maud Ravary, Jean-Louis Goeb, François Medjkane, Jean Xavier, on behalf of The Therapeutic Body Wrap Study group

**Affiliations:** 1 Département de Psychiatrie de l’Enfant et de l’Adolescent, Centre Hospitalier Universitaire de Lille, Lille, France; 2 Université de Lille, Centre Hospitalier Universitaire de Lille, Equipe d’Accueil A2694—Santé publique: épidémiologie et qualité des soins, Lille, France; 3 Université de Lille, Inserm, Centre Hospitalier Universitaire de Lille, CIC1403—Centre d'investigation clinique, Lille, France; 4 Département de Psychiatrie de l’Enfant et de l’Adolescent, Groupe Hospitalier Pitié-Salpêtrière, APHP, Paris, France; 5 CNRS UMR 7222 Institut des Systèmes Intelligents et Robotiques, Université Pierre & Marie Curie, Paris, France; 6 Pôle 59i03, Département de Psychiatrie de l’Enfant et de l’Adolescent, Etablissement Public de Santé Mentale Lille-Métropole, Armentières, France; IRCCS E. Medea, ITALY

## Abstract

**Introduction:**

The use of therapeutic body wraps (TBW) has been reported in small series or case reports, but has become controversial.

**Objectives:**

This is a feasibility, multicentre, randomized, controlled, open-label trial with blinded outcome assessment (PROBE design).

**Setting:**

Children with autism and severe-injurious behaviours (SIB) were enrolled from 13 specialized clinics.

**Interventions:**

Dry-sheet TBW (DRY group) vs. wet-sheet TBW (WET group).

**Primary outcome measures:**

3-month change in the Aberrant Behaviour Checklist irritability score (ABC-irritability) within per-protocol (PP) sample.

**Results:**

From January 2008 to January 2015, we recruited 48 children (age range: 5.9 to 9.9 years, 78.1% male). Seven patients (4 in the DRY group, 3 in the WET group) were dropped from the study early and were excluded from PP analysis. At endpoint, ABC-irritability significantly improved in both groups (means (standard deviation) = -11.15 (8.05) in the DRY group and -10.57 (9.29) in the WET group), as did the other ABC scores and the Children Autism Rating scale score. However, there was no significant difference between groups. All but 5 patients were rated as much or very much improved. A repeated-measures analysis confirmed the significant improvement in ABC-irritability scores according to time (p < .0001), with no significant difference between the two groups (group effect: p = .55; interaction time x group: p = .27). Pooling both groups together, the mean 3-month change from baseline in ABC-irritability score was -10.90 (effect size = 1.59, p < .0001).

**Conclusions:**

We found that feasibility was overall satisfactory with a slow recruitment rate and a rather good attrition rate. TBW was a safe complementary therapy in this population. There was no difference between wet and dry TBW at 3 months, and ABC-irritability significantly decreased with both wet and dry sheet TBW. To assess whether TBW may constitute an alternative to medication or behavioural intervention for treating SIB in ASD patients, a larger randomized comparative trial (e.g. TBW vs. antipsychotics) is warranted.

**Trial registration:**

ClinicalTrials.gov NCT03164746.

## Introduction

Treatment of severe auto/hetero aggressive behaviours in children and adolescents with autism spectrum disorder (ASD) is a complex issue. It is useful to treat symptoms as well as underlying psychiatric conditions or behavioural dysfunction. Symptomatic treatments include behavioural and family interventions and psychotropic medications, mostly sedative drugs, mood stabilizers and antipsychotics. To date, only a few atypical antipsychotics have been approved for minors to treat irritability and behavioural impairment associated with intellectual disability and/or pervasive developmental disorder, and such drugs have numerous adverse effects [1]. In some resistant cases, clozapine [2], intensive behavioural intervention [3], electro-convulsive therapy (ECT) [4–5] or inpatient stays with a multidisciplinary approach [6] have been recommended, even in children and adolescents. The search for other therapeutic options is urgent.

Therapeutic body wraps (TBW), also called wet sheet packs or packing therapy, are an adjunct treatment administered by nurses or occupational therapists. Typically, TBW sessions last 45-min. TBW involves enveloping a patient in damp sheets during the session while he or she is invited to express him/herself [7]. First evidence of its use with children can be found in a German textbook from the mid-19^th^ century in which TBW belonged to so-called hydrotherapy [8]. Among early scientific publications in adult patients, we found a case of delirium tremens successfully managed with wet sheet pack [9], and a series of 1000 warm TBW administered to patients with ‘manic depressive illness’ and ‘dementia praecox’ and found a ‘quieting effect’ in most cases [10]. In young patients, Leahy and Sands [11] reported on the first series of children treated with dry or wet TBW for behavioural symptoms following Von Economo epidemic encephalitis (with wet TBW being better for severe cases, according to the authors). In the era of efficient treatment (e.g., antipsychotics; ECT) for adult psychosis, TBW tended to become anecdotal. More recently, TBW was used in adult psychiatric inpatients as an alternative to physical constraints [12] or to control anxiety [13]. Interestingly, in the largest recent retrospective study, which included 172 cases, TBW showed potential to reduce the dose of both anxiolytic and antipsychotic drugs [13].

In children and adolescents, there are two main indications. First, TBW has been proposed in moderate to severe atopic dermatitis [14–15]. When it is integrated in a multidisciplinary treatment program, it appears to be a safe and efficient treatment [16–17]. Second, TBW has been proposed as an adjunct treatment for severe injurious behaviours in borderline adolescents [18–19], in catatonia [20–21] and in children and adolescents with ASD [18–22]. The largest series reported so far was an open clinical reporting on a sample of 10 outpatients aged 5 to 16 years with ASD and severe behavioural impairments; these patients showed significant improvement on the Aberrant Behavior Checklist (ABC) after TBW was implemented. The total ABC scores and irritability and hyperactivity sub-scores decreased 38%, 50% and 42%, respectively [22].

Due to the absence of empirical studies supporting TBW, its usefulness with children and adolescents with ASD has become controversial, mainly in France [23–25]. French regulation authorities asked for clinical studies to be performed to define whether TBW should be continued on the basis of relevant scientific research [26]. The current study aimed to evaluate the feasibility of comparing wet TBW versus dry TBW in children and adolescents with ASD and severe injurious behaviours in the context of a single-blind randomized controlled trial [27]. Based on the sensory integration (SI) theory [28, 29], we hypothesized that if an improvement occurred with TBW, it would be significantly better with a wet sheet pack than a dry sheet pack. SI is the hierarchical organization of somatic sensations that occurs during development: as sensory experiences occur, SI develops within adaptation responses, which are defined as a purposeful, goal-directed response to sensory experiences. SI serves as foundations for an individual’s perceptions, behaviours and learning. All senses (auditory, vestibular, proprioceptive, tactile and visual) are progressively integrated as a *body percept* and are rooted in different psychosomatic functions (e.g. the coordination of the two sides of the body, activity level, attention span, motor planning and emotional stability). SI dysfunction results in a wide variety of developmental disorders [30], including ASD. Wet TBW should mainly activate two sensory experiences–pressure/proprioception and temperature/tactile sense–whereas dry TBW should activate only the proprioception route.

## Methods

### Study design

This is a multicentre, single-blind, randomized, two-arm, controlled, feasibility study, with balanced randomization in two parallel groups (wet TBW versus dry TBW). In its initial version, the study was planned to assess the efficacy of wet TBW versus dry TBW and versus risperidone. Given the French authorities’ expectation to collect safety data and the reluctance of some parents toward risperidone use, the protocol was amended in accordance with French authorities’ decision to provide data about the effect size and safety of wet TBW versus dry TBW. This change subsequently allowed a sharp decrease in the number of participants. The risperidone arm was abandoned and it was not possible to add another arm with patients exposed to another non-pharmacological intervention (e.g. applied behavioural analysis, ABA) because randomization would have been changed dramatically and the duration of the study to reach enough statistical power would have been even longer. As a consequence, the study became a feasibility study after this amendment [27]. The ethics committee validated all these amendments to the protocol before breaking the blindness, including the validation of the final statistical analysis plan.

The randomization procedure was centralized according to a randomization table provided by the sponsor. The computer-generated randomization table allowed a 1:1 allocation using a block size of six. The block size had been blinded for investigators. The randomization list was not disclosed to the study centres, monitors, project statisticians or project team. Allocation was concealed using a sealed opaque envelope system and occurred as soon as the inclusion was decided. Since maintaining blindness to treatment was not possible with therapists in charge of the TBW because of wet/dry sheets, we used independent raters to assess primary and secondary variables (see list below) following the PROBE (Prospective Randomized Open Blinded End-point) design.

### Participants

From January 2008 to January 2015, children were recruited from 13 French specialized clinics (*Centre Hospitalier Universitaire* in Lille, *Institut Départemental Albert Calmette* in Camiers, *Centre Hospitalier Robert Ballanger* in Aulnay-sous-Bois, *Groupe Hospitalier Pitié-Salpêtrière* in Paris, *Centre Hospitalier de Douai* in Douai, *Etablissement Public de Santé Mentale Val de Lys-Artois* in Saint-Venant, *Centre de Santé Mentale Angevin* in Angers, *Institut Médico-éducatif* in Montigny in Ostrevent, *Centre Hospitalier de Lens* in Lens, *Etablissement de Santé Maison Blanche* in Paris, *Etablissement Public de Santé Roger Prévot* in Gennevilliers, *Centre Hospitalier Montfavet* in Avignon, and *Centre Hospitalier d’Arras* in Arras) under the supervision of the Department of Child and Adolescent Psychiatry, Lille University Hospital, Lille, France. The study was registered at the French Drug Agency (AFSSAPS) under the number 2007-A01376-47. The study was then approved by both the Lille University Hospital ethics committee (*Comité de Protection des Personnes CPP Nord Ouest IV* under agreement number CCP 08/08) and the French Ministry of Health (number DGS 2008–0070). All these regulatory requirements were made before first patient’s enrolment. As non-medication trials are not publicly available in AFSSAPS registry and are registered in French, the clinical trial was also registered in ClinicalTrials.gov registry under the number NCT03164746. The authors confirm that all ongoing and related trials for this drug/intervention are registered.

**Fig 1:**
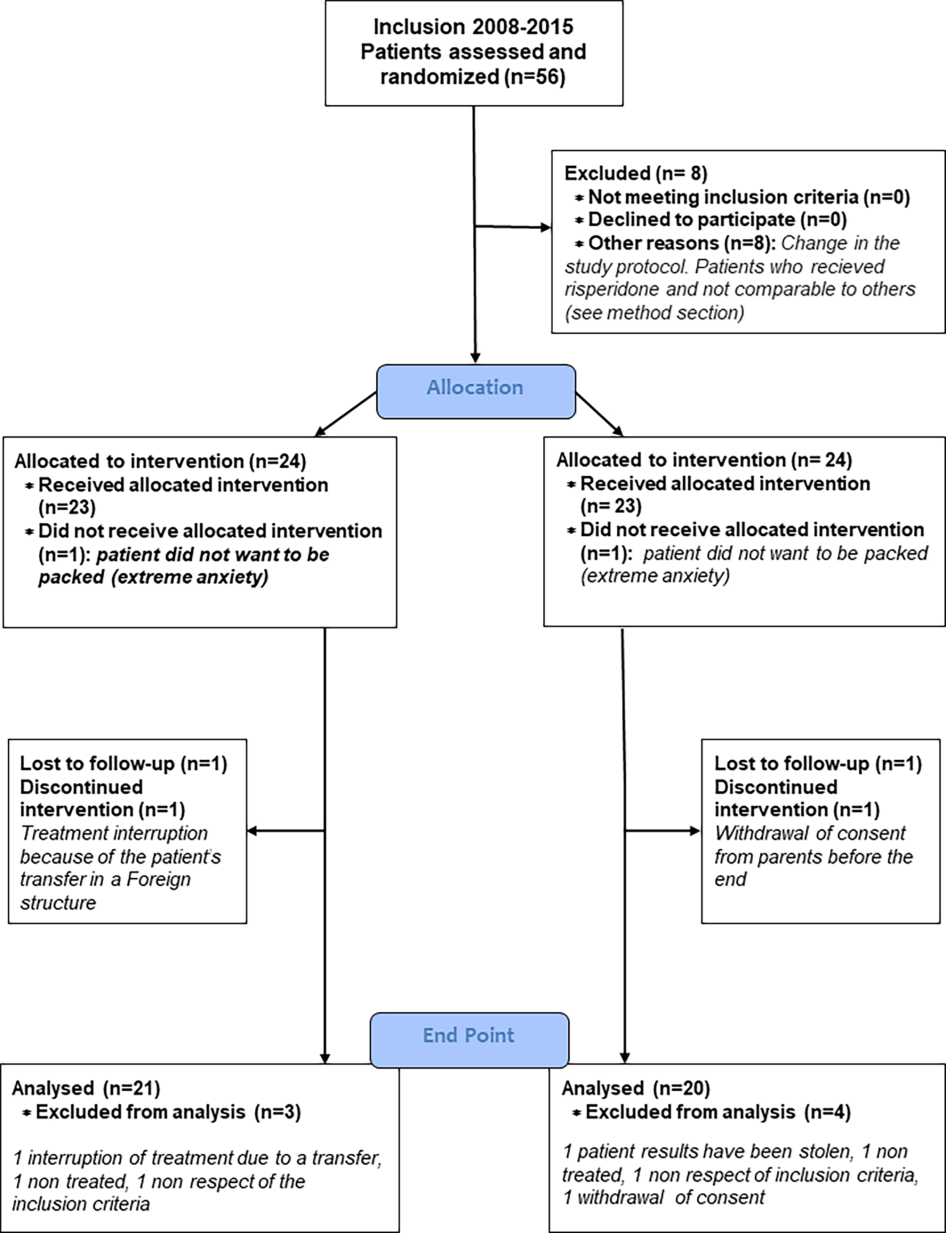
CONSORT diagram flow of the study.

Each parent (and child, when possible) gave informed written consent before inclusion. Inclusion criteria were: a current diagnosis of autism, Asperger syndrome, atypical autism according to ICD-10 criteria [31] confirmed by specialized clinical assessment, being aged 5 to 18 years, and presenting severe behavioural disturbances such as hetero and self-injurious behaviours, automutilations, severe motor hyperactivity, and severe stereotypies. We excluded children with known organic syndrome and/or non-stabilized neuropaediatric (e.g., seizures) or medical (e.g., diabetes mellitus) comorbidities. This was systematically checked after a neuropaediatric assessment. For patients with a stabilized seizure condition, antiepileptic medication had to be stable for at least 4 weeks. Also, for patients already given psychotropic medications, the prescription had to be stable for at least 4 weeks. In total, 56 patients were eligible and assessed at baseline. They all were randomized to a study arm, including 8 patients randomized in the risperidone arm. Finally, 48 entered the trial to receive TBW and were allocated to the WET group (N = 24) or the DRY group (N = 24) (see Fig 1: CONSORT flow diagram).

### Intervention: Therapeutic body wrap

TBW is aimed at restoring sensory integration and body representations and reducing anxiety. The overall treatment encompassed a series of twice-a-week sessions over 3 months. The sessions took place in the same quiet room, and they usually lasted 45 minutes each, though they could be extended up to 1 hour depending on the patient’s response. A minimum of 30 minutes of TBW inside the sheets is recommended. During the sessions, the patient wore a bathing suit. The sessions were conducted by an occupational therapist (*psychomotricien* in the French medical system) and involved at least two members of the patient’s care team that were asked to keep on participating to all patient’s sessions. All occupational therapists that offered TBW were trained previously by the Lille team, the principal investigator of the study. At the beginning of the session, the patient’s consent to proceed was orally obtained, as no session was compulsory. In case of non-language, therapists checked behavioural manifestations of refusal. Then, the patient was first wrapped in wet damp sheets (cold phase) and covered up with a rescue and a dry blanket. Afterward, the body spontaneously warmed up (warm phase). The patient was then invited to freely express his feelings, bodily/cutaneous sensations and somatic fantasies. A brief moment (usually less than 15 minutes) of drawing or modelling with clay was proposed at the end of each session to provide non-verbal avenues through which the patient could express feelings and explore body representations. Throughout the session, the patient’s comments and relevant observations from the clinicians (e.g., clinical signs, body image, cenesthesis sensations, and adverse events) were carefully recorded by one of the observers [7,20]. A 10-minute-video presents video clips of the same child during several sessions both at session beginnings and session endings (available at http://doi.org/10.5281/zenodo.1157306). The clinical history of this child has been reported in [32]. The intervention was similar in the dry sheet pack control group, with the notable exception that the sheets were dry during all sessions. Apart common training with the coordination team, to ensure comparability between the 13 clinical centers, occupational therapists in charge of TBW sessions received regular supervision on sites (4 times a year) by the coordination team.

### Clinical measures

Autism symptom dimensions were assessed at baseline using the current Autism Diagnostic Interview-revised (ADI-R), addressed to parents [33]. To assess clinical change during the 3-month exploratory study, we used a double-blind procedure, and we measured the primary variable at enrolment, 1 month, 2 months and 3 months. The primary outcome variable was the Aberrant Behavior Checklist (ABC) irritability score [34]. Secondary variables included: (1) the other ABC scores: hyperactivity, lethargy, inappropriate speech, stereotypic behaviour and total; (2) the Clinical Global Impression-Severity (CGI-S) scale at enrolment and the Clinical Global Impression-Improvement (CGI-I) scale at 3 months [35]; and (3) the Child Autism Rating Scale (CARS) [36] at enrolment and at 3 months. For each instrument, French translation sources are given in supporting information.

### Statistical analysis

After the protocol was amended, the study was designed as a feasibility study. The primary objective was the comparison of change in ABC-irritability scores from baseline to 3 months between the two groups (wet TBW *vs*. dry TBW). According to the recruitment potential, we planned to recruit 30 subjects in each group. This sample size would allow us to detect a minimum effect size (Cohen’s d: difference in means divided by the root mean square standard deviations) of 0.74 between the 2 groups for the comparison of mean changes in irritability score from baseline to 3 months with a power of 80% (two-sided test and type I error of 5%); this effect size was considered as large in the literature [37].

Categorical variables were expressed as frequencies and percentages. Continuous variables were expressed as the means (standard deviation) in the case of a normal distribution and as medians (inter quartiles) otherwise. The normality of distributions was checked graphically and using the Shapiro-Wilk test. For categorical variables, the baseline characteristics were described for each group and compared using a chi-square test or Fisher’s exact test. For continuous variables, we used Student’s t test or the Mann-Whitney U test when appropriate. The magnitude of the between-group differences was assessed by calculating the absolute standardized differences (Cohen’s d: difference in means or rates divided by the root mean square standard deviations); an absolute standardized difference >0.20 was interpreted as a meaningful difference.

A comparison of primary outcomes between the 2 groups was performed with an analysis of covariance (ANCOVA), adjusted for the baseline value. The effect size (namely, the baseline-adjusted mean differences in 3-month change from baseline divided by the root mean square error of ANCOVA model) was computed taking into account the adjustment for baseline, and its 95% confidence interval was estimated using a bootstrap resampling. The validity of the ANCOVA model was checked by examining the model residuals. The same methodology was employed for the secondary outcomes. When the normality of model residuals was not satisfied, nonparametric analysis was used. The relative change between baseline and the 3-month visit was calculated and compared between the 2 groups using the Mann-Whitney U test.

Analysis of repeated measurements (months 1, 2 and 3) for the primary and secondary outcomes was performed using the linear mixed model (LMM). LMM allows handling the correlations between the repeated measurements. These correlations were modelled using a covariance pattern model. In this study, we chose a first-order, autoregressive covariance pattern (because the measurements were taken at predetermined and evenly spaced intervals). In this model, the effects were baseline, time, group and group-by-time interaction. The correlations between continuous variables were analysed using Spearman’s rank correlation coefficient. A post-hoc exploratory analysis was performed on the primary and secondary outcomes by pooling the two groups together. The change between baseline and 3 months was analysed using the paired Student’s t test in the case of a normal distribution and the Wilcoxon signed-rank test otherwise. The effect sizes were computed as the mean differences (3-months—baseline) divided by standard deviations at baseline. All statistical tests were performed at the 2-tailed α level of 0.05. The data were analysed using SAS version 9.4 [SAS Institute Inc., Cary, NC 27513, USA].

## Results

### Participants’ characteristics and adverse events

Of the 48 patients randomized and allocated to TBW, 7 patients (3 in the WET group and 4 in the DRY group) dropped out of the study early (see CONSORT diagram flow in Fig 1). The reasons for dropout included the patient’s refusal (extreme anxiety) to be packed (n = 2, 1 in each group); the patient’s transfer to a different location (n = 1); withdrawal of consent from parents before the study ended (n = 1); non-respect for the inclusion criteria (n = 2); and lost data (n = 1). Apart from the two patients who dropped out because they refused to be wrapped due to severe anxiety, no other serious adverse events related to the trial procedure occurred. In total, 5 severe adverse events were reported to the French authorities: 2 severe temper outbursts from the same subject; 1 fall with head trauma, suspected to be related to epilepsy; 1 case of an asthma attack; and 1 accidental fall that left the patient with several bone fractures. None of these adverse events were considered related to the procedure after imputability was checked by French authorities.

Given patients’ profiles, many– 10 (41.67%) and 13 (54.17%) in the WET and DRY groups, respectively–had been receiving medication at the protocol baseline. A description of the treatment at baseline and eventual changes during the trial are summarized in Table 1. All antipsychotics dosages are given in mg of equivalent chlorpromazine (EqChlor). At baseline, there was no difference in the number of patients receiving medication. However, we found a difference between mean EqChlor in patients receiving an antipsychotic medication (WET: mean = 110.50 mg, n = 10; DRY:mean = 186.50 mg, n = 13). The most frequent medication was risperidone but most patients receiving drug treatment had poly-medications and received off-label drugs (e.g. clozapine). For all but 3 patients, the prescriptions remained stable during the study. Two patients in the WET group and 1 patient in the DRY group had their prescription modified. All changes occurred at the 1-month visit and remained stable at the 2- and 3-month visits. Haloperidol (4 mg/day) and tropatepine were added to clozapine (300 mg/day) for one patient. Topiramate (25 mg/day) replaced valproate (250 mg/day) for the second patient. This patient also received lithium carbonate (400 mg/day) for a brief period (less than 15 days). Finally, the last patient kept the same drugs, but minor changes in the respective dosages occurred: he received 60 mg EqChlor more of the total antipsychotics.

**Table 1:** Medications of the participants at baseline.

	WET (N = 24)	DRY (N = 24)
**At baseline**		
Patients not on medication	n = 14	n = 11
Patients receiving medication (*Poly-medication*)	n = 10 (*n = 6*)	n = 13 (*n = 10*)
Equivalent chlorpromazine (mg) per patient receiving medication	110.50 mg	186.50 mg
**During the trial**		
Not on medication	n = 14	n = 11
No change in medication	n = 8	n = 12
Change in medication	n = 2 (8%)	n = 1 (4%)
Change in equivalent chlorpromazine in total	+ 60 mg	+ 200 mg
**List of compounds**		
Antipsychotics (*risperidone*)	n = 14 (*n = 7*)	n = 19 (*n = 9*)
Anti-seizures	n = 2	n = 0
Melatonin	n = 1	n = 1
Antiparkinsonian	n = 0	n = 1
Benzodiazepine	n = 3	n = 2
Other drugs	n = 4	n = 0

In total, the per-protocol analysis included 41 children (age: mean (SD) = 8.30 (2.80), range: 5.90 to 9.90 years, 78.05% male) with ASD: 20 were randomized to dry TBW (DRY group), and 21 were randomized to wet TWB (WET group). Participants’ characteristics are summarized in Table 2.

**Table 2:** Socio-demographic and clinical characteristics of the participants at baseline that were included in the per-protocol analysis.

	WET (N = 21)	DRY (N = 20)	*p (ASDiff)*
**Demographics**)			
Age, mean (SD	7.71 (2.58)	8.86 (3.08)	0.20 (0.40)
Male–female (% male) (*)	17–4 (80.95%)	15–5 (75%)	0.72 (0.14)
**ADI-R**			
Social domain, mean (SD)	24.70 (4.83)	24.0 (6.38)	0.71 (0.12)
Communication, mean (SD)	13.10 (2.82)	13.10 (4.86)	0.98 (0.01)
Stereotypies, mean (SD)	7.60 (2.46)	6.10 (2.74)	0.06 (0.60)
**Diagnosis**	Autism: N = 7	Autism: N = 5	NA
	Atypical autism: N = 13	Atypical autism: N = 14	
	Asperger: N = 1	Asperger: N = 1	
**ABC scores at baseline**			
Irritability, mean (SD)	28.57 (7.03)	29.20 (6.69)	0.77 (0.09)
Hyperactivity, mean (SD)	33.26 (9.25)	36.53 (5.50)	0.20 (0.43)
Lethargy, mean (SD)	25.74 (8.97)	19.89 (13.16)	0.12 (0.52)
Stereotypic behaviour, mean (SD)	12.11 (4.09)	9.95 (6.45)	0.23 (0.40)
Inappropriate speech, median (Q1 to Q3)**	0 (0 to 5)	0 (0 to 2)	0.08 (0.49)
Total	94.29 (27.99)	98.20 (27.69)	0.66 (0.14)
**CARS at baseline,** mean (SD)	46.29 (6.63)	42.08 (8.53)	0.09 (0.55)
**CGI-Severity,** mean (SD)	6.42 (0.67)	6.26 (0.81)	0.51 (0.22)

ABC: Aberrant Behavior Checklist; CARS: Child Autism Rating Scale; CGI-S: Clinical Global Impression-Severity; NA: not applicable; Q1 (Q3): first (third) quartile; ASDiff: absolute standardized difference; comparisons were performed using Student’s t test unless otherwise specified

*Fisher’s exact test

**Mann-Whitney U test.

### Primary variable outcome

**Fig 2:**
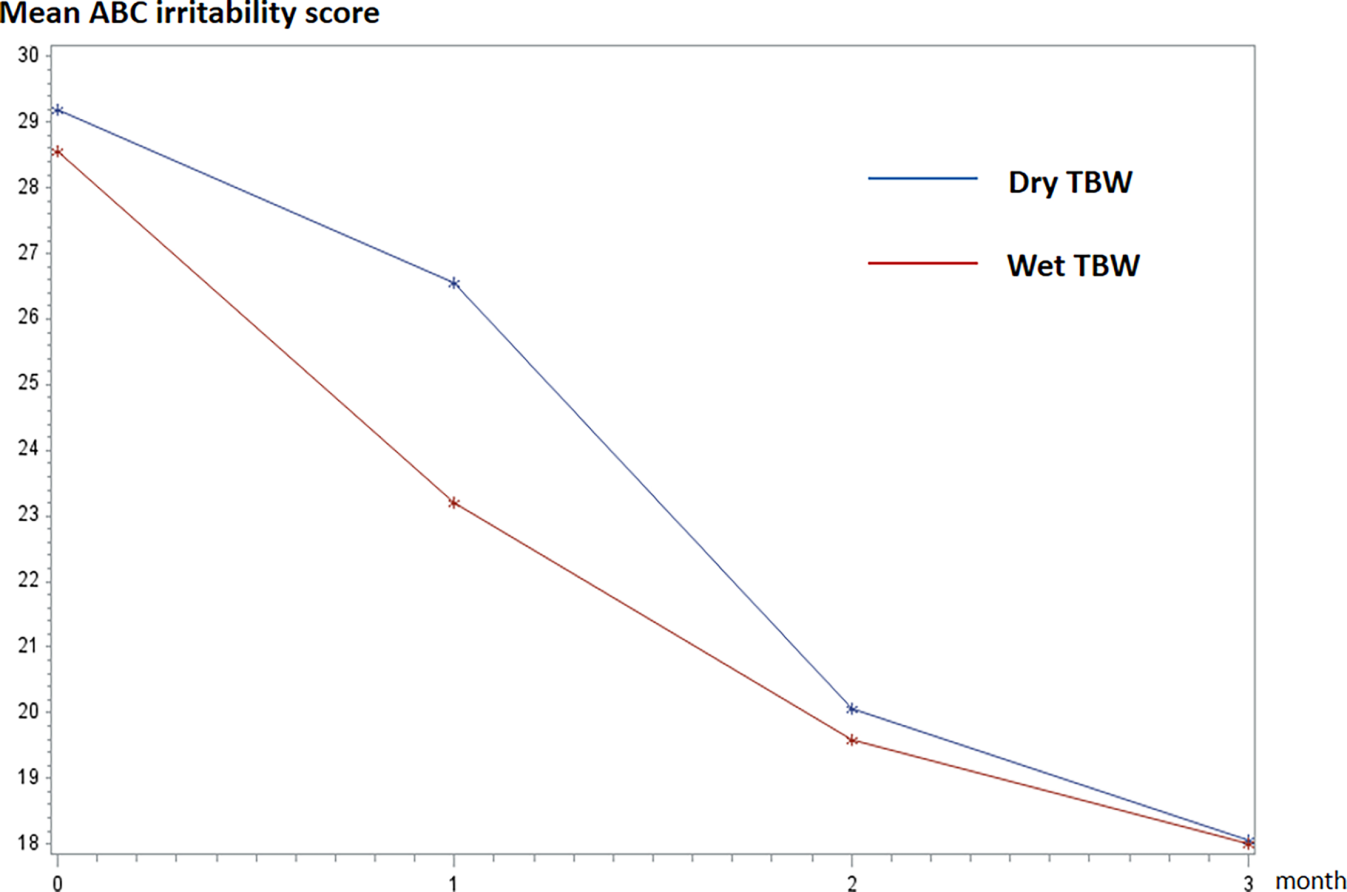
Course of the mean ABC-irritability score over the 3-month study duration for wet TBW (red) and dry TBW (blue).

Table 3 summarizes the primary and secondary outcomes (change from baseline to 3 months). We found in both groups an improvement in ABC-irritability scores at the endpoint (mean change (standard deviation) = -11.15 (8.05) in the DRY group and -10.57 (9.29) in the WET group). However, there was no significant difference between the experimental (wet TBW) group and the control (dry TBW) group (adjusted mean differences (95% CI): -0.17 (-4.99 to -4.64); p = .94). The effect size (ES: baseline-adjusted mean differences in change divided by the root mean square error) between the two groups was near zero (ES = 0.03: 95% CI: 0.019 to 0.22). Fig 2 shows the course of ABC-irritability scores over the 3-month study duration. Linear mixed models analysing the repeated measures confirmed the significant improvement over time (p < .0001), while these improvements were not significantly different between the groups (time-by-group interaction: p = .27), and no significant global group effect was found (p = .55).

**Table 3:** Change from baseline to 3 months in primary and secondary outcomes (3-month value minus baseline value).

Outcomes	WET (N = 21)	DRY (N = 20)	Effect size**	P*
	N; (3-month—baseline)	N; (3-month—baseline)		
ABC-Irritability, mean (SD)	21; -10.57 (9.29)	20; -11.15 (8.05)	0.03	0.94
ABC-Hyperactivity, mean (SD)	19; -13.47 (8.65)	19; -12.89 (9.46)	0.31	0.36
ABC-Lethargy, mean (SD)	19; -10.16 (7.41)	19; -6.84 (7.32)	0.10	0.76
ABC-Stereotypic behaviour, mean (SD)	19; -3.05 (2.86)	19; -2.32 (2.86)	0.06	0.87
ABC-Inappropriate speech, median (Q1 to Q3)	19; 0 (-1 to 0)	19; 0 (-2 to 0)	NA	0.7
ABC-Total, mean (SD)	20; -36.55 (20.57)	20; -35.0 (28.21)	0.08	0.80
CARS, mean (SD)	17; -5.03 (4.37)	17; -4.18 (3.07)	0.14	0.69
CGI-I, median (Q1 to Q3)	19; -1 (-2 to -0.50)	18; -1 (-1 to -0.50)	NA	0.36

ABC: Aberrant Behavior Checklist; CARS: Child Autism Rating Scale; CGI-I: Clinical Global Impression-Improvement; NA: not applicable; SD: standard deviation; Q1 (Q3): first (third) quartile

* p-value adjusted for baseline values using analysis of covariance (ANCOVA), except for ABC-Inappropriate speech and CGI-I, for which Mann-Whitney U tests were used

**: baseline-adjusted mean differences in change divided by the root mean square error of ANCOVA model.

We found no significant correlation between age and change in ABC-irritability score in the dry TBW (Spearman = -0.09; p = 0.70) or wet TBW (Spearman = 0.20; p = 0.39) or between the number of sessions and change in the primary variable in dry TBW (Spearman = 0.27; p = 0.24) or wet TBW (Spearman = -0.17; p = 0.47).

### Secondary outcomes and secondary analyses

The analysis of the secondary variables (hyperactivity-, lethargy-, inappropriate speech-, stereotypic behaviour- and total ABC scores; CARS) displayed a profile similar to the primary outcome variable. We found in both groups an improvement in all secondary variables except the ABC-inappropriate speech sub-score. However, these improvements were not significantly different between groups (Table 3). At 3 months, we found 12/18 (66.67%) much improved and 3/18 (16.670%) very much improved patients in the dry TBW group, and we found 13/19 (68.42%) much improved and 6/19 (31.58%) very much improved patients in the wet TBW group (chi-square test, p = 0.91 and Fisher's Exact test, p = 0.29, respectively).

Since we found no difference between groups, no significant correlation between ABC-irritability changes and age and no significant correlation between ABC-irritability changes and the number of TBW sessions, a post-hoc exploratory analysis was performed by pooling the two groups to estimate the effect of TBW (whether wet or dry sheets were used) for each variable at 3 months. The effect sizes are given in Table 4. All variables, except the ABC-inappropriate speech subscore, significantly improved after 3 months of biweekly TBW sessions, with effect sizes ranging from 0.49 to 1.76.

**Table 4:** Change from baseline to 3 months in primary and secondary outcomes in the pooled sample (N = 41).

Outcomes	Baseline	3-months N; values	Between-group differences	Effect size**	P *
	N; values	N; values	N; values		
			(3-months—baseline)		
ABC-Irritability, mean (SD)	41; 28.88 (6.79)	41; 18.02 (7.79)	41; -10.85 (8.60)	1.60	< .0001
ABC-Hyperactivity, mean (SD)	38; 34.89 (7.69)	38; 21.71 (8.63)	38; -13.18 (8.94)	1.72	< .0001
ABC-Lethargy, mean (SD)	38; 22.81 (11.50)	38; 14.32 (7.73)	38; -8.50 (7.46)	0.74	< .0001
ABC-Stereotypic behaviour, mean (SD)	38; 11.02 (5.44)	38; 8.34 (4.86)	38; -2.68 (4.47)	0.49	0.0007
ABC-Inappropriate speech, median (Q1 to Q3)	38; 0 (0 to 5)	38; 0 (0 to 3)	38; 0 (-1 to 0)	NA	0.35
ABC-Total, mean (SD)	41; 96.16 (27.56)	40; 62.18 (25.42)	40; -35.78 (24.38)	1.30	< .0001
CARS, mean (SD)	40; 44.29 (7.79)	34; 39.62 (7.89)	34; -4.60 (3.74)	0.59	< .0001
CGI-I, median (Q1 to Q3)	38; 6.53 (6 to 7)	37; 5 (5 to 6)	37; -1 (-1.50 to -.5)	1.76	< .0001

ABC: Aberrant Behavior Checklist; CARS: Child Autism Rating Scale; CGI-I: Clinical Global Impression-Improvement

*Paired Student's t test except for ABC-Inappropriate speech (Wilcoxon signed-rank test); SD: standard deviation; Q1 to Q3: first to third quartile

**mean difference (3 months—baseline) divided by standard deviation at baseline.

## Discussion

### Summary of the results

We aimed to evaluate the feasibility of comparing wet TBW versus dry TBW in children and adolescents with ASD and severe injurious behaviours in the context of a single-blinded randomized controlled trial. The sample recruited was remarkable for the severity of the patients (e.g. ASD+SIB, severity scores at baseline, 50% taking medication). We found that feasibility was overall satisfactory despite a slow recruitment rate in the context of an increasing French controversy regarding TBW. However, we had a rather good attrition rate and few severe adverse events, meaning that the procedure was well tolerated. Based on sensory integration theory [28–29], we hypothesized that if an improvement occurred with TBW, it would be significantly better with wet sheet pack than dry sheet pack, as wet TBW activates two sensory experiences–pressure/proprioception and temperature/tactile sense–whereas dry TBW should activate only the proprioception route. The results did not support our hypothesis. There was no difference between wet and dry TBW at 3 months, and ABC-irritability significantly decreased with both wet and dry sheet TBW. To assess whether TBW may constitute an alternative to medication (or behavioural intervention) for treating SIB in ASD patients, a larger randomized comparative trial (TBW vs. antipsychotics) is warranted. The recruitment of patients, naïve from medication, would be more appropriate to adequately assess the efficacy of TBW versus medication.

### Therapeutic body wrap compared with other treatment for severe behavioural impairment in ASD

Challenging behaviours in ASD are complex clinical issues that have been addressed through diverse treatment proposals. Two second generation antipsychotics–risperidone and aripiprazole–have received specific FDA approvals for behavioural impairments (self-injury, aggression and agitation) associated with autism and/or ID in children and adolescents (age 6–17 years) [1]. However, a meta-analysis showed that adverse events with second generation antipsychotics were numerous and under-estimated in children and adolescents [38]. Apart from medications, ABA and associated intensive behavioural interventions are efficient in relieving self-injurious and aggressive behaviours [3]. First-line treatment models combine psychopharmacological and behavioural assessment and treatment development. They are particularly effective in evaluating the contributing roles of environmental, or operant, functions of challenging behaviours, along with underlying psychotropic-responsive psychiatric conditions [39].

Unfortunately, challenging behaviour may persist in some patients despite exhaustive interdisciplinary interventions that expose both patients and caregivers to significant injury risk and psychosocial morbidity. Only case reports or series are currently available regarding other therapeutic approaches for extreme behavioural conditions, considered by some as highly controversial, such as electroconvulsive therapy (ECT) [5,40] and/or TBW [18,22]. The feasibility randomized control trial reported here is the first evidence from a substantial sample that TBW may be an option for relieving behavioural dysfunction in these challenging patients. The results did not distinguish wet vs. dry TBW, meaning that the two options are similar in terms of patients’ response. Interestingly, the strong effect size at 3 months was in the same range as the one found in Goeb [22] smaller open study. However, the formal efficacy of TBW should be explored in a randomized trial with an active treatment arm (e.g. aripiprazole or risperidone).

Several teams have proposed receiving the most severe cases in neurobehavioural units dedicated to resistant acute situations associated with autism and/or ID [41]. These units were modelled after a similar unit in the USA (http://www.kennedykrieger.org/patient-care/patient-care-programs/inpatient-programs/neurobehavioral-unit-nbu, 2014-10-17), and they aim at defining all risk factors associated with these challenging behaviours. They pursue a multimodal framework for the acute evaluation and treatment of these challenging conditions, and they tailor an individual approach based on targeted risk factors. Guinchat et al. [6] recently reported the largest series. Most common aetiologies for acute behavioural crises were organic causes (including epilepsy and painful medical conditions), environmental causes (including lack of treatment and adjustment disorder), and non-ASD psychiatric conditions (including catatonia, major depressive episode, bipolar disorder, and schizophrenia). Causes with specific treatment were associated with better outcomes (e.g., painful medical condition or non-ASD psychiatric diagnosis), justifying the tailored approach. Additionally, longer hospitalization was associated with better outcomes even after adjusting for confounding factors [6]. Cravero et al. [42] reported the case of a young adult with Cornelia de Lange syndrome and Ehlers-Danlos syndrome that summarizes the multidisciplinary neurobehavioural inpatient approach. The patient presented nonverbal autism, intellectual disability and severe/intractable self-harming behaviours that led to a life-threatening complication (e.g., septicaemia). A significant reduction in self-harming behaviours was attained after addressing all causes of somatic pains, managing pain using level II and III analgesics, stabilizing the patient’s mood, limiting the iatrogenic effects of multiple prescriptions and offering a specific psycho-educational approach.

Summarizing the literature, it appears that treatment of challenging behaviours in individuals with ASD should be classified in the following way: symptomatic treatment (e.g., antipsychotics, or ABA reinforcing approach of more appropriate behaviours) or specific treatment targeting a given risk factor (e.g., treatment of painful medical condition, ABA treatment of deviant operant communication, or ECT in case of intractable depression). Whether it is provided with wet or dry sheets, TBW should be included among the adjuvant symptomatic approaches. In contrast to its use in atopic dermatitis, where some research suggests that wet TBW may limit local inflammation, improve skin hydration and favour local effects of corticoids on skin lesions [43,44], we still do not know how TBW alleviates behavioural impairments among individuals with ASD and/or ID. Given the lack of differences found between wet and dry TBW, we speculate a specific role of body pressure that is related to proprioception.

### Limitations

The results of the current study should be interpreted in the context of its limitations. First, the study did not include an arm to compare TBW with risperidone (the only compounds that have a French authority approval for behavioural impairments in children with ASD and/or ID). This was due to the change in the protocol requested by the French regulatory agency in response to the controversies surrounding TBW unethical use [23] and antipsychotics’ secondary effects [38]. Given the slow recruitment, French authority asked for stopping the risperidone arm and changing the objective of the study form efficacy to feasibility, in order to shorten the timing of the first results. Second, the per-protocol sample size was limited to 41 individuals, excluding a secondary analysis on clinical factors involved in ASD heterogeneity. Third, many participants (48%) received medication. Although the prescription regimen remained stable for all except 3 patients, we cannot exclude that medication affected interpretation of the results. Also, we cannot exclude that the experience of TBW may be influenced by taking concomitant medications. Forth, mild secondary effects are difficult to capture in patients with very severe autism, ID and no language. Therefore, the overall good tolerance of the treatment might be underestimated. However, in the field of atopic dermatitis, in which approximately 200 young participants have been reported, wet TBW are also well tolerated and most reported secondary effects relate to specific skin problems and combined corticoid treatment (e.g., folliculitis) [15].

## Conclusion

We investigated the use of TBW in children with SIB associated with ASD in the context of a feasibility study. We found that feasibility was overall satisfactory with a slow recruitment rate and a rather good attrition rate. Both wet and dry sheet TBWs were overall well tolerated. Regarding clinical response, we did not find any differences between wet and dry TBW at 3 months. However, ABC-irritability scores significantly decreased, and TBW provided with both wet and dry sheets had a significant similar effect size. Therefore, given the absence of significant adverse events, TBW should be investigated to ensure its efficacy. To assess whether TBW may constitute an alternative to medication for treating SIB in ASD patients, a larger randomized comparative trial (TBW vs. antipsychotics) is warranted. The recruitment of naïve patients would be more appropriate to adequately assess the efficacy of TBW versus medication.

## Supporting information

S1 File Clinical.gov registration form. (PDF)

S2 File Ethics committee study agreement (*Comité de Protection des Personnes CPP Nord Ouest IV*). (PDF)

S3 File French ministry of health study agreement (*Direction General de la Santé*). (PDF)

S4 File French ministry of health study request (*Direction General de la Santé*). (PDF)

S5 File CONSORT checklist. (PDF)

S6 File Video clips of the same child during several TBW sessions both at session beginnings and session endings. The video is available at http://doi.org/10.5281/zenodo.1157306.
(PDF)

S7 File Study instruments and French translation sources. (PDF)

S8 File Study protocol French original version. (PDF)

S9 File Study protocol English translated version. (PDF)
