# Supplementary material for: Therapeutic body wraps (TBW) for treatment of severe injurious behaviour in children with autism spectrum disorder (ASD): A 3-month randomized controlled feasibility study
Source: PLoS One. 2018 Jun 29;13(6):e0198726. doi: 10.1371/journal.pone.0198726 (PMC6025870; doi:10.1371/journal.pone.0198726)
Supplement: S1 File — (PDF) [file pone.0198726.s001.pdf]

ClinicalTrials.gov Protocol Registration and Results System (PRS) Receipt
Release Date: May 22, 2017

ClinicalTrials.gov ID: NCT03164746

Study Identification

Unique Protocol ID: 2007/0715

Brief Title: Treatment by Therapeutic Body Wraps in Children and Adolescents Suffering From Autism With Severe Injurious Behavior. (PACKING)

Official Title: Demonstration of the Effectiveness of Treatment by Therapeutic Body Wraps in Children and Adolescents Suffering From Autism Spectrum Disorder With Severe Injurious Behavior.

Secondary IDs: 2007-A01376-47 [ID-RCB number, ANSM]
DGS 2008-0070 [DGS number]

Study Status

Record Verification: May 2017

Overall Status: Completed

Study Start: December 2007 []

Primary Completion: December 2014 [Actual]

Study Completion: December 2014 [Actual]

Sponsor/Collaborators

Sponsor: University Hospital, Lille

Responsible Party: Sponsor

Collaborators: Ministry of Health, France

Oversight

U.S. FDA-regulated Drug: No

U.S. FDA-regulated Device: No

U.S. FDA IND/IDE: No

Human Subjects Review: Board Status: Approved
Approval Number: CPP 08/08
Board Name: Comité de Protection des Personnes Nord Ouest IV
Board Affiliation: Agence Régionale de Santé (ARS) Nord Pas-de-Calais
Phone: 3 20 44 41 65
Email: cppnordouestiv@univ-lille2.fr
Address:

Bâtiment ex USN B (RDC)

6 rue du Professeur Laguesse
CHRU LILLE CS 70001
59 037 LILLE CEDEX

Data Monitoring: Yes

Plan to Share IPD: Undecided

FDA Regulated Intervention: No

Study Description

Brief Summary: Severe injurious behaviors in children with autism spectrum disorder are challenging. First line treatment approaches include behavioral techniques but pharmacotherapy is frequently required despite frequent adverse effects in youths.

Therapeutic body wraps has been reported in small series or case reports, but has never been assessed in the context of a randomized controlled trial.

The present study is an exploratory, multicenter, randomized, controlled, open label with blinded outcome assessment (PROBE design) trial of the effect of wet versus dry therapeutic body wraps in children presenting with autism spectrum disorder and severe injurious behavior.

Detailed Description: Packing therapy has never been assessed, namely in children with severe injurious behavior and autism spectrum disorder.

The aim of the present study is to evaluate the beneficial effect of wet versus dry therapeutic body wraps through an exploratory randomized controlled open label blinded outcome assessment approach.

The primary objective is the comparison of change in ABC irritability scores from baseline to 3 months between the two groups. According to the potential recruitment, we plan to recruit 30 subjects in each group. This sample size could allow us to detect a minimum effect size of 0.74 between the 2 groups (considered large in literature) with a power of 80% (two-sided test and type I error of 5%).

As described elsewhere, wet or dry session will be organized through twice-a-week sessions for a 3-month duration.

Comparison in primary outcome (ABC irritability score) between the 2 groups will be performed using Analysis of Covariance (ANCOVA) adjusted for the baseline value. The standardized difference (effect size) will be computed taking into account the adjustment for baseline and its 95% confidence interval will be estimated using a bootstrap resampling. The validity of the ANCOVA model will be checked by examining the model residuals.

The same methodology will be used for the secondary outcomes.

Conditions

Conditions: Autism Spectrum Disorder

Keywords: Autism
Therapeutic body wraps

Study Design

Study Type: Interventional

Primary Purpose:	Supportive Care
Study Phase:	N/A
Interventional Study Model:	Parallel Assignment
Number of Arms:	2
Masking:	Investigator, Outcomes Assessor
Allocation:	Randomized
Enrollment:	48 [Actual]

Arms and Interventions

Arms	Assigned Interventions
Active Comparator: DRY group<br>Dry sheet therapeutic body wraps will be conducted through twice-a-week sessions for a 3-month duration. Sessions take place in the same quiet room and they usually last 45 minutes each up to 1 hour depending on the patient's response. During sessions, the patient wearied a bathing suit. Sessions were conducted under the supervision of an occupational therapist and involved at least two members of the patient's care team.	Dry sheet Therapeutic body wraps<br>At the beginning of the session, the patient's consent to proceed was orally obtained, as no session was compulsory. The therapists checked behavioral manifestations of refusal. Then, the patient was first wrapped in Dry damp sheets (cold phase) and covered up with a rescue and a dry blanket. Afterward the body spontaneously warmed up (warm phase). The patient was then invited to freely express his feelings.
Experimental: WET Group<br>Wet sheet therapeutic body wraps will be conducted through twice-a-week sessions for a 3-month duration. Sessions take place in the same quiet room and they usually last 45 minutes each up to 1 hour depending on the patient's response. During sessions, the patient wearied a bathing suit. Sessions were conducted under the supervision of an occupational therapist and involved at least two members of the patient's care team.	WET sheet Therapeutic body wraps<br>At the beginning of the session, the patient's consent to proceed was orally obtained, as no session was compulsory. The therapists checked behavioral manifestations of refusal. Then, the patient was first wrapped in wet damp sheets (cold phase) and covered up with a rescue and a dry blanket. Afterward the body spontaneously warmed up (warm phase). The patient was then invited to freely express his feelings.

Outcome Measures

Primary Outcome Measure:

1. Aberrant Behavior Checklist (ABC) irritability score
   Measure the reduction in the intensity of the behavioral disorders objectified by the sub-score Irritability of the scale ABC

   [Time Frame: Baseline and 3 months]

Secondary Outcome Measure:

2. ABC hyperactivity score
   Measure the reduction in the intensity of the behavioral disorders objectified by the sub-score hyperactivity of the scale ABC

   [Time Frame: Baseline,1-month, 2-month and 3-months]
3. ABC lethargy score
   Measure the reduction in the intensity of the behavioral disorders objectified by the sub-score lethargy of the scale ABC

   [Time Frame: Baseline,1-month, 2-month and 3-months]
4. ABC inappropriate speech score

Measure the reduction in the intensity of the behavioral disorders objectified by the sub-score inappropriate speech of the scale ABC

[Time Frame: Baseline,1-month, 2-month and 3-months]

5. ABC stereotypic behavior score
   Measure the reduction in the intensity of the behavioral disorders objectified by the sub-score stereotypic behavior of the scale ABC

   [Time Frame: Baseline,1-month, 2-month and 3-months]
6. ABC Total score
   Measure the reduction in the intensity of the behavioral disorders objectified by the total score of the scale ABC

   [Time Frame: Baseline,1-month, 2-month and 3-months]
7. Child Autism Rating Scale (CARS)
   Measure the decrease in the intensity of autistic symptoms

   [Time Frame: Baseline,1-month, 2-month and 3-months]
8. Clinical Global Impression-Improvement (CGI-I)
   Measure the global clinical outcome

   [Time Frame: Baseline,1-month, 2-month and 3-months]
9. Clinical Global Impression-Severity (CGI-S)
   Measure the global clinical outcome

   [Time Frame: Baseline,1-month, 2-month and 3-months]

Eligibility

Minimum Age: 5 Years

Maximum Age: 18 Years

Sex: All

Gender Based: No

Accepts Healthy Volunteers: No

Criteria: Inclusion Criteria:

- a current diagnosis of autism, Asperger syndrome, atypical autism according ICD-10 criteria confirmed by specialized clinical assessment;
- presenting severe behavioural disturbances such as hetero and self-injurious behaviours, automutilation, severe motor hyperactivity, severe stereotypies.
- having a systematically consultation by a neuro pediatric.

Exclusion Criteria:

- children with known organic syndrome and/or non-stabilized neuropediatric (e.g. seizures) or medical (e.g. diabetes mellitus) comorbidities.
- patients with stabilized seizure condition, antiepileptic medication should be stable for at least 4 weeks.

Contacts/Locations

Central Contact Person: Pierre Delion, MD,PhD
Email: pierre.delion@chru-lille.fr

Central Contact Backup: Dominique Deplanque, MD, PhD
Email: dominique.deplanque@chru-lille.fr

Study Officials: Pierre Delion, MD, PhD
Study Principal Investigator
University hospital Lille

Locations: France
Hôpital Fontan, CHRU
Lille, France

Institut Départemental Albert Calmette
Camiers, France

Centre Hospitalier Robert Ballanger
Aulnay-sous-Bois, France

Groupe Hospitalier Pitier-Salpêtrière
Paris, France

Centre Hospitalier de Douai
Douai, France

Etablissement Public de Santé Mentale Val de Lys-Artois
Saint-Venant, France

Centre de Santé Mentale Angevin
Angers, France

Institut Médico-éducatif
Montigny-en-Ostrevent, France

Centre Hospitalier de Lens
Lens, France

Etablissement de Santé Maison Blanche
Paris, France

Etablissement Public de Santé Roger Prévot
Gennevilliers, France

Centre Hospitalier Montfavet
Avignon, France

Centre Hospitalier d'Arras
Arras, France

References

Citations:

Links:

Study Data/Documents:
