# Supplementary material for: Therapeutic body wraps (TBW) for treatment of severe injurious behaviour in children with autism spectrum disorder (ASD): A 3-month randomized controlled feasibility study
Source: PLoS One. 2018 Jun 29;13(6):e0198726. doi: 10.1371/journal.pone.0198726 (PMC6025870; doi:10.1371/journal.pone.0198726)
Supplement: S2 File — (PDF) [file pone.0198726.s002.pdf]

Comité de Protection des Personnes Nord Ouest IV

Président : J.P. JOUET
Vice-Président : X. LABBEE
Secrétaire : J.Ch. ARCHANGE
Trésorier : Y. VENDEL

Membres titulaires :
F. ASKEVIS-LEHERPEUX
R. BEUSCART
S. COSTA
A. De BOUVET
M. JOLY
R. MATIS
P. ODOU
Y. TOMME
L. WILLIATTE-PELLITTERI

Membres suppléants :
B. CORTET
D. DECAMPS-MINI
M. DE MEDEIROS
S. DUHEM
P. HANNEQUART
A. LECOCQ
P. MACIAG
G. MARCHAL
C. SULMAN
C. THERY
E. TORCK-BAUMELOU
F. VASSEUR

C.H.R.U. LILLE
Délégation à la Recherche
27 MAI 2008

Lille, le 20 mai 2008

Mr FIEVE
Délégation à la Recherche
Administration Générale
CHRU de LILLE

Mr le Dr JL GOEB
Hôpital FONTAN
CHRU de LILLE

COMPTE RENDU DE DELIBERATION

Référence à rappeler dans toute correspondance : CPP 08/08
Intitulé du projet : *« Démonstration de l'efficacité des traitements par packing chez les enfants et adolescents souffrant de troubles autistiques avec troubles graves du comportement. »*
Promoteur : CHRU de Lille
Investigateur principal : Dr GOEB
Référence de la version reçue : Version 5 du protocole datée du 20 avril 2008
Note d'information et formulaire de consentement version 5 datée du 20 avril 2008
N° EudraCT : 2007-A01376-47

Date de la réunion : Mardi 13 mai 2008
Membres titulaires présents :
Collège n°1 :
Mr R. BEUSCART – Représentant des Personnes Qualifiées en Recherche Biomédicale
Mme S. COSTA – Représentant des Infirmiers
Mr J.P. JOUET - Représentant des Personnes Qualifiées en Recherche Biomédicale
Mme Y. VENDEL - Représentant des Personnes Qualifiées en Recherche Biomédicale
Mr R. MATIS - Représentant des Personnes Qualifiées en Recherche Biomédicale
Collège n°2 :
Mme F. ASKEVIS-LEHERPEUX – Représentant des Psychologues
Mme A. De BOUVET – Représentant des Personnes qualifiées en matière d'éthique
Mme L. WILLIATTE-PELLETERI - Représentant des Personnes Qualifiées en matière juridique
Mme M. JOLY – Représentant d'association agréée de malades ou d'usagers du système de santé
Mme V. BARON – Représentant des Travailleurs Sociaux
Mr TOMME - Représentant d'association agréée de malades ou d'usagers du système de santé

Secrétariat :
S. Duriez
Service de Pharmacologie
Faculté de Médecine
Pôle Recherche
1 place de Verdun
59045 LILLE Cedex

Tel : 03 20 44 54 49
Fax : 03 20 44 68 63
Email : sduriez@univ-lille2.fr

Monsieur le Directeur, Cher Confrère,

Le Comité de Protection des Personnes Nord Ouest IV, lors de sa réunion du mardi 13 mai 2008, a pris connaissance des documents concernant le projet cité en référence.

Outre les documents étudiés, un document audiovisuel a été projeté aux membres du Comité, expliquant le traitement par packing.

Le Comité de Protection des Personnes Nord Ouest IV émet un **AVIS FAVORABLE** à la menée de cette étude.

Il souhaiterait simplement que les centres investigateurs soient plus clairement identifiés et déclarés.

Cette étude est conforme aux articles L1121-1 et L1123-7 du code de la santé publique définissant les conditions de validité de la recherche.

Cet avis a été rendu sans que les membres éventuellement concernés par 'étude aient pris part au vote.

Je vous prie de croire, Monsieur le Directeur, Cher Confrère, à l'expression de mes meilleures salutations.

Pr JP. JOUET
Président du CPP Nord Ouest IV
