# Supplementary material for: Therapeutic body wraps (TBW) for treatment of severe injurious behaviour in children with autism spectrum disorder (ASD): A 3-month randomized controlled feasibility study
Source: PLoS One. 2018 Jun 29;13(6):e0198726. doi: 10.1371/journal.pone.0198726 (PMC6025870; doi:10.1371/journal.pone.0198726)
Supplement: S3 File — (PDF) [file pone.0198726.s003.pdf]

O.H.R.U. LILLE
Délégation à la Recherche

- 9 MAI 2008

Liberté • Égalité • Fraternité
RÉPUBLIQUE FRANÇAISE

O.H.R.U. LILLE
Direction Générale

0 9 MAI 2008

ARRIVÉE

Ministère de la Santé, de la Jeunesse, des Sports et de la Vie associative

Direction générale de la santé
Sous-Direction de la politique des pratiques
et des produits de santé
Bureau de la qualité des pratiques
et des recherches biomédicales
DGS / PP1 / RBM 08.297

Paris, le -5 MAI 2008

CHU DE LILLE
Monsieur le Directeur Général
2, avenue Oscar Lambert
59037 LILLE CEDEX

Personne chargée du dossier :
Mme le Dr. Sabine KENOUCH
Tél. : 01 40 56 57 33
Fax : 01 40 56 56 55
E-mail : sabine.kenouch@sante.gouv.fr

N° d'enregistrement : 2007-A01376-47

Monsieur le Directeur Général,

Par votre courrier du 04/02/2008, reçu le 21/02/2008, vous m'avez adressé, en qualité de promoteur ou de représentant du promoteur, une demande d'autorisation de mise en œuvre de la recherche biomédicale suivante :

Démonstration de l'efficacité des traitements par packing chez les enfants et adolescents souffrants de troubles autistiques avec troubles graves du comportement
Investigateur coordonnateur : Jean-Louis GOEB

Je vous informe que l'autorisation de mise en œuvre de cette recherche biomédicale vous est accordée sous le numéro : DGS2008-0070

En outre, je vous rappelle qu'en application de l'article L. 1123-6 du code de la santé publique, cette recherche ne pourra commencer qu'après qu'un Comité de protection des personnes aura émis un avis favorable sur le projet.

Pour toute demande de modification substantielle, toute déclaration d'effets indésirables graves inattendus, toute déclaration de fin de recherche ou toute autre correspondance relative à cette recherche, il conviendra de rappeler le numéro d'enregistrement mentionné en haut de la présente lettre.

Veuillez agréer, Monsieur le Directeur Général, l'expression de ma considération distinguée

La Sous-directrice de la politique
des pratiques et des produits de santé

Catherine LEFRANC
