# Supplementary material for: Therapeutic body wraps (TBW) for treatment of severe injurious behaviour in children with autism spectrum disorder (ASD): A 3-month randomized controlled feasibility study
Source: PLoS One. 2018 Jun 29;13(6):e0198726. doi: 10.1371/journal.pone.0198726 (PMC6025870; doi:10.1371/journal.pone.0198726)
Supplement: S4 File — (PDF) [file pone.0198726.s004.pdf]

DEMANDE D'AUTORISATION AUPRES DU MINISTRE CHARGE DE LA SANTE ET DEMANDE D'AVIS AU COMITÉ DE PROTECTION DES PERSONNES POUR UNE RECHERCHE BIOMEDICALE AUTRE QUE CELLES PORTANT SUR LES PRODUITS VISES A L'ARTICLE L. 5311-1 DU CODE DE LA SANTE PUBLIQUE

Partie réservée à l'autorité compétente (AC) / au Comité de protection des personnes (CPP) :

Date de réception de la demande :	Date de demande d'informations complémentaires :	Autorisation de l'AC:<br>☐oui ☐non<br>Date :
Date d'enregistrement de la demande considérée complète :<br><br>Date du début de la procédure :	Date de réception des informations complémentaires / amendées :	Avis du CPP :<br>☐favorable ☐défavorable<br>Date :

Partie à compléter par le demandeur :

Ce formulaire est commun pour la demande d'autorisation auprès de l'autorité compétente et pour la demande d'avis au comité de protection des personnes.

DEMANDE D'AUTORISATION A L'AUTORITE COMPETENTE :	Oui
DEMANDE D'AVIS AU CPP :	

A. IDENTIFICATION DE LA RECHERCHE BIOMEDICALE

État membre dans lequel la demande est soumise : FRANCE

Titre complet de la recherche	Démonstration de lefficacité des traitements par packing chez les enfants et adolescent souffrant de troubles autistiques avec troubles grave du comortement

Numéro d'enregistrement de la recherché délivré par l'AFSSAPS : 2007-A01376-47

Numéro de code du protocole de la recherche donné par le promoteur,	Version	Date
2007/0715	version 4	29/11/2007

Nom ou titre abrégé de la recherche biomédicale, le cas échéant :

S'agit-il d'une recherche initialement qualifiée de "soins courants" Non

Inscription au fichier VRB Non

B. IDENTIFICATION DU PROMOTEUR RESPONSABLE DE LA DEMANDE

B1. Promoteur	
-Nom de l'organisme :	CHU DE LILLE
-Nom de la personne à contacter :	Aurélie GOZDZIASZEK
-Adresse :	2, avenue Oscar Lambert LILLE CEDEX 59037
-Numéro de téléphone :	
-Numéro de télécopie :	
-Courriel :	

B2. Représentant légal du promoteur dans la Communauté européenne pour la recherche biomédicale (si différent du promoteur)	
-Nom de l'organisme :	Centre Hospitalier Régional et Universitaire de LILLE
-Nom de la personne à contacter :	Mr Régis Fiévé
-Adresse :	Délégation à la Recherche
-Numéro de téléphone :	03 20 44 41 45
-Numéro de télécopie :	03 20 44 57 11
-Courriel :	drc@chru-lille.fr

Statut du promoteur : non commercial

C. IDENTIFICATION DU DEMANDEUR

C1. Demande pour l'autorité compétente

-Promoteur

-Nom de l'organisme :	CHU DE LILLE
-Nom de la personne à contacter :	Aurélie GOZDZIASZEK
-Adresse :	2, avenue Oscar Lambert LILLE CEDEX 59037
-Numéro de téléphone :	
-Numéro de télécopie :	
-Courriel :	

C2. Demande pour le Comité de protection des personnes

-Promoteur

-Nom de l'organisme :	
-Nom de la personne à contacter :	
-Adresse :	
-Numéro de téléphone :	
-Numéro de télécopie :	
-Courriel :	

G. INFORMATIONS GÉNÉRALES SUR LA RECHERCHE

Condition médicale ou pathologie étudiée
Préciser la condition médicale :Autisme
Classification CIM : 10
Classification MedDRA :

Est-ce une maladie rare ?	non

Objectif(s) de la recherche
Objectif principal :

mesurer lefficacité à 3 mois des techniques de packing dans les troubles graves du comportement des enfants présentant un TED en tant que traitement adjuvant de la prescription de rispéridone

Objectifs secondaires :

Evaluer la place des packings dans la stratégie thérapeutique globale à proposer aux enfants autistes les plus gravement malades

Principaux critères d’inclusion (énumérer les plus importants)

· Enfants et adolescent(e)s âgés de plus de 5 ans présentant selon les critères internationaux (CIM 10) évalués par lADI-R : un syndrome autistique, un syndrome dAsperger, ou un trouble envahissant du développement non spécifique ; et nécessitant un tra

Principaux critères de non inclusion (énumérer les plus importants)

· Sujets présentant un syndrome de Rett ou un trouble désintégratif de lenfance ;
· sujets présentant une contre-indication à la rispéridone ou présentant des antécédents de syndrome malin aux neuroleptiques ;
· absence daccord parental après informat

Critère(s) d’évaluation principal(aux)

Diminution de lintensité des troubles du comportement objectivés par le sous-score Irritabilité de léchelle Aberrant Behavior Checklist (ABC) à la 12ème semaine (critère principal).

Domaine(s) d’étude

- stratégies thérapeutiques et préventives

Méthodologie de la recherche

Tirage au sort :	oui
la recherche comporte-t-elle une comparaison de groupes?	oui

Préciser le(s) comparateur(s) utilisé(s) :	
- (d') autre(s) produits(s)	non
- placebo	non
- autre	oui
Si oui, préciser : seviettes sèches	

la recherche est-elle multicentrique?	oui
la recherche est-elle prévue pour être menée dans plusieurs Etats membres ?	non
Cette recherche implique-t-elle des pays tiers ?	non

Durée maximale de participation pour un sujet selon le protocole :

3mois

Définition de la fin de la recherche et justification, si celle-ci ne correspond pas à la date de la dernière visite de la dernière personne participant à la recherche :

Estimation initiale de la durée de la recherche :

-en France :	3 ans 0 mois
-dans tous les pays concernés par la recherche :	0 ans 0 mois

H. PERSONNES PARTICIPANT A LA RECHERHE

Age

Tranche d’âge étudiée	
	< 18 ans
	Enfants (2-11 ans)
	Adolescents (12-17 ans)

Sexe

Femmes	Hommes

Personnes participant à la recherche

Volontaires sains		non
Malades	oui	
- femmes enceintes		non
- femmes allaitantes		non
- femmes parturientes		non
- personnes privées de liberté		non
- personnes en situation d’urgence		non
- personnes incapables de donner personnellement leur consentement	oui	
dont majeurs sous tutelle		non

Nombre prévu de personnes à inclure :

- en France : 128

En cas de recherche menée dans plusieurs pays :

- dans la Communauté européenne : 0

- pour l’ensemble des pays participant à la recherche :

I. INVESTIGATEURS ET LIEUX DE RECHERCHE

I.1. Investigateur coordinateur
Nom :GOEB
Prénoms :Jean-Louis

N°ADELI :591189576

Qualification, spécialité: PH Psychiatre
Adresse: CHRU Lille, Hôpital Fontan Service du Pr DELLION 6, rue Émile Verhaeghe Lille 59037<br>Courriel: jlgoeb@wanadoo.fr, jl-goeb@chru-lille.fr

I.2. Autres investigateurs

Nom : MILLE
Prénoms : Christian
ADELI : 801010489
Qualification, spécialité : Professeur de psychiatrie de l'enfant et de l'adolescent
Adresse : Consultation de psychopathologie de l'enfant et de l'adolescent,CHRU Amiens Hôpital Nord - Place V Plauchet
Courriel : mille.christian@chu-amiens.fr

I.2. Autres investigateurs

Nom : BALEYTE
Prénoms : Jean-Marc
ADELI : 141045666
Qualification, spécialité : Pr de psychiatrie
Adresse : service de psychiatrie de lenfant et de ladolescent, au CHRU Clemenceau, Caen
Courriel : baleyte-jm@chu-caen.fr

J. INFORMATION SUR LE COMITE DE PROTECTION DES PERSONNES (CPP)

Nom et adresse : CPP NORD-OUEST IV
CHU Faculté de Médecine Pôle Recherche LILLE Cedex 59045

avis :	à demander

K. SIGNATURE DU DEMANDEUR EN FRANCE

Par la présente, j’atteste / j’atteste au nom du promoteur (rayer la mention inutile) ce qui suit :

- les informations fournies ci-dessus à l’appui de la demande sont exactes ;
- la recherche biomédicale sera réalisée conformément au protocole, à la réglementation nationale et aux principes de bonnes pratiques cliniques ;
- il est raisonnable d’entreprendre la recherche biomédicale proposée ;
- je soumettrai un résumé du rapport final de la recherche à l’autorité compétente et au comité de protection des personnes concerné au plus tard 1 an après la fin de la recherche dans tous les pays ;
- je déclarerai la date effective du commencement de la recherche au Ministre chargé de la Santé et au comité de protection des personnes concerné dès qu’elle sera connue.

DEMANDEUR auprès de l’autorité compétente (comme indiqué à la section C.1) :<br>Date :<br>Signature :<br>Nom :	DEMANDEUR auprès du CPP (comme indiqué à la section C.1) :<br>Date :<br>Signature<br>Nom:
