# Supplementary material for: Therapeutic body wraps (TBW) for treatment of severe injurious behaviour in children with autism spectrum disorder (ASD): A 3-month randomized controlled feasibility study
Source: PLoS One. 2018 Jun 29;13(6):e0198726. doi: 10.1371/journal.pone.0198726 (PMC6025870; doi:10.1371/journal.pone.0198726)
Supplement: S6 File — The video is available at http://doi.org/10.5281/zenodo.1157306. (PDF) [file pone.0198726.s006.pdf]

Supporting information S6

Video clips of the same child during several Therapeutic Body Wrap sessions both at session beginnings and session endings.

The video is available at http://doi.org/10.5281/zenodo.1157306

The clinical history of this child has been reported in Cravero C, Guinchat V, Xavier J, Meunier C, Diaz L, Mignot C, Doummar D, Chantot-Bastaraud S, Consoli A, Cohen D. Management of severe developmental regression in an autistic child with a 1q21.3 microdeletion and self-injurious blindness. Case Rep Psychiatry 2017; 2017: e7582780.

Authorization was given by the child’s mother.
