# Supplementary material for: Therapeutic body wraps (TBW) for treatment of severe injurious behaviour in children with autism spectrum disorder (ASD): A 3-month randomized controlled feasibility study
Source: PLoS One. 2018 Jun 29;13(6):e0198726. doi: 10.1371/journal.pone.0198726 (PMC6025870; doi:10.1371/journal.pone.0198726)
Supplement: S7 File — (PDF) [file pone.0198726.s007.pdf]

Supporting information S7 : Study instruments and French translation sources

The ***Child Autism Rating Scale*** (CARS) was published in 1986 in an English version by Eric Schopler team (Schopler et al., 1986). It was translated into French by Bernadette Rogé and published in 1989. To our knowledge, there was no validation performed in French but the scale has been widely use in French and is recommended by the French regulatory authorities to assess patients with autism (HAS, 2012). The ***Autism Diagnostic Interview-Revised*** was published in 1994 by Lord et al. in 1994. The French version was translated by Bernadette Rogé, Eric Fombonne, Jeanne Fremolle-Kruck and Evelyne Arti. It is available through Hogrefe publisher that indicates that the validation was performed on a sample of 70 individuals. The ***Aberrant Behavior Checklist*** (ABC) has been used in several studies conducted by pharmaceutical companies on behavioral impairment in children and adolescent with autism or intellectual disability (Aman et al., 1985). Both Janssen (for risperidone studies) and Otsuka (for aripiprazole studies) have translated and validated the French version of the ABC. To our knowledge, validation have not been made publicly available. However, consistency of its value to show changes in behaviors with efficient treatment has been repetitively shown in both medication and behavioral studies including studies with French, Canadian, Swiss or Belgium sites (HAS, 2012). However, since 2005, a national prospective cohort has been promoted and longitudinal data are now available on ADI-R, CARS and ABC showing good descriptive values and high correlation between scores (Yanni-Coudurier et al., 2016). Finally, the ***Clinical Global Impression-Severity*** (CGI-S) and ***Clinical Global Impression-Improvement*** (CGI-I) scales have been used in many studies both in longitudinal studies and pharmacological trials. The French version was translated by Marc Ansseau in the 80ties (Guelfi et al., 1993).

Aman MG, Singh NN, Stewart AW, & Field CJ. The aberrant behavior checklist: a behavior rating scale for the assessment of treatment effects. Am J Ment Defic 1985; 89(5): 485– 491.

Guelfi JD (ed). L'évaluation clinique standardisée en psychiatrie. Editions médicales Pierre Fabre. Castres, 1993 (2 tomes).

Guy W. Clinical global impression scale. The ECDEU Assessment Manual for Psychopharmacology-Revised 1976; Volume DHEW Publ No ADM 76, 338: 218–222.

Rogé B. Adaptation Française de l'échelle d'évaluation de l'autisme infantile (C.A.R.S). Issy-les-Moluineaux : Editions d'Applications psychotechniques, 1989.

Schopler E, Reichler RJ, Renner BR. The Childhood Autism Rating Scale (CARS). Los Angeles, CA 1986; Western Psychological Services.

Lord C, Rutter M, Le Couteur A. Autism Diagnostic Interview-Revised: a revised version of a diagnostic interview for caregivers of individuals with possible pervasive developmental disorders. J Autism Dev Disord 1994; 24(5): 659-85.

Rogé B, Fombonne E, Fremolle-Kruck J, Arti E. ADI-R: Entretien semi-structuré pour le diagnostic de l'autisme. 2011; http://www.hogrefe.fr/produit/adi-r-entretien-semi-structure-pour-le-diagnostic-de-l-autisme/#.WmglVuf7lPZ

HAS. Autisme et autres troubles envahissants du développement: interventions éducatives et thérapeutiques coordonnées chez l'enfant et l'adolescent. Argumentaire Scientifique, 2012; Mars, 1–60. http://doi.org/978-2-11-128519-4.

Yanni-Coudurier C, Rattaz C, Baghdadli A. Facteurs liés à l'évolution des compétences adaptatives chez 77 jeunes enfants avec troubles du spectre autistique. Neuropsychiatr Enf 2016; 64: 367-375.
