# Supplementary material for: Therapeutic body wraps (TBW) for treatment of severe injurious behaviour in children with autism spectrum disorder (ASD): A 3-month randomized controlled feasibility study
Source: PLoS One. 2018 Jun 29;13(6):e0198726. doi: 10.1371/journal.pone.0198726 (PMC6025870; doi:10.1371/journal.pone.0198726)
Supplement: S8 File — (PDF) [file pone.0198726.s008.pdf]

DEMONSTRATION DE L'EFFICACITE DES TRAITEMENTS PAR PACKING CHEZ LES ENFANTS SOUFFRANTS DE TROUBLES AUTISTIQUES AVEC TROUBLES GRAVES DU COMPORTEMENT

Investigateur Coordinateur :

Pr Pierre Delion
Service de Psychiatrie de l'Enfant et de l'Adolescent
du Pr Pierre DELION,
Hôpital Michel Fontan,
Centre Hospitalier Universitaire de Lille

Investigateurs principaux

Pr Jean-Marc BALEYTE,
Service de Psychiatrie de l'Enfant et de l'Adolescent,
Centre Hospitalier Universitaire de Caen

Pr Christian MILLE,
Service de Psychiatrie de l'Enfant et de l'Adolescent,
Centre Hospitalier Universitaire d'Amiens

Co-Investigateurs

CHU de LILLE : Dr Jean-Louis GOEB, Dr Anne-Yvonne LENFANT, Dr Renaud JARDRI, Dr Frank BONELLI, Dr Géraldine KECHID.

Promoteur de l'étude

CHRU de Lille

2, avenue Oscar Lambret

59037 Lille Cedex

II – DATE ET SIGNATURES

Identité	Date	Signature
Dr Jean Louis GOEB		
Pr DELION		
Dr Anne-Yvonne LENFANT,		
Dr Renaud JARDRI		
Dr Frank BONELLI		
Dr Géraldine KECHID		
Pr Christian MILLE		
Pr Jean Marc BALEYTE		

III - SOMMAIRE

I PAGE DE TITRE *1*

II DATE ET SIGNATURES *2*

III SOMMAIRE *3*

IV RESUME DU PROJET *5*

V JUSTIFICATION DE L'ETUDE *6*

1.1 Autismes et Troubles Envahissants du Développement (TED) 6

1.2 Traitements psychotropes 6

1.3 Packing 6

VI OBJECTIF(S) DE L'ETUDE 8

A) Objectif principal 8

B) Objectifs secondaires 8

C) Critère principal de jugement 8

D) Critères secondaires de jugement 8

VII DESCRIPTION DE L'ETUDE *9*

A) Méthodologie 9

B) Organisation générale de l'étude : 9

VIII DESCRIPTION DES SUJETS *12*

A) Calcul du nombre de sujets nécessaires 12

B) Critères d'inclusion 12

C) Critères de non-inclusion 13

D) Bénéfices attendus pour le sujet 13

E) Critères d'arrêt de l'étude pour un sujet qui y participe 13

IX TRAITEMENTS *13*

X BIOLOGIE 13

XI EVENEMENTS INDESIRABLES GRAVES *14*

A) Définition 14

B) Evenements indésirables et risques prévisibles liés au protocole 14

C) Déclaration 15

XII METHODES D'ANALYSE DES PARAMETRES MESURES 15

XIII DUREE DE L'ETUDE *16*

XIV RESPONSABILITES (LEGALES ET PRATIQUES) *16*

A) Moyens nécessaires 16

B) Type de participation attendu des différents acteurs 17

C) Soumission pour avis au CPP et pour autorisation à la DGS 18

D) Amendements au protocole 18

E) Information des sujets et recueil écrit du consentement éclairé 18

F) Cahiers d'observation 19

G) Assurance qualité 19

XV CONFIDENTIALITE **20**

XVI ASSURANCE **20**
XVII CONVENTIONS FINANCIERES **20**
XVIII RAPPORT FINAL ET PUBLICATIONS **20**
XIX RESPONSABILITES DE L'INVESTIGATEUR **21**
XX ANNEXES **21**

IV – RESUME DU PROJET

RESUME DE L'ETUDE

Promoteur	CHRU de LILLE
Justification	Certains enfants et adolescents présentant un syndrome autiste présentent des troubles graves du comportement mal contrôlés par leur prise en charge habituelle. La prise en charge par packing donne des résultats cliniques précieux qu'il convient d'établir scientifiquement afin de préciser la place de ce soin dans le cadre d'une prise en charge globale de l'enfant (thérapeutique, éducative et pédagogique).
Objectifs	Mesurer l'efficacité à trois mois des techniques de packing dans les troubles graves du comportement des enfants présentant un TED<br>Un objectif secondaire est d'évaluer la place des packing dans la stratégie thérapeutique globale à proposer aux enfants autistes les plus gravement malades.
Plan de l'étude	Essai thérapeutique Phase II.
Effectif	40 sujets seront recrutés (2 groupes de 20 sujets).
Critères d'inclusion	Enfants et adolescents âgés de plus de 3 ans présentant selon les critères internationaux (CIM 10) évalués par l'ADI-R : un syndrome autistique, un syndrome d'Asperger, ou un trouble envahissant du développement non spécifique ;<br>- présentant des troubles graves du comportement (tels que auto ou hétéro-agressivité, automutilations, instabilité psychomotrice sévère, stéréotypies graves et envahissantes) ;<br>- ayant bénéficié d'une consultation neuropédiatrique ;<br>- pour les patients épileptiques : posologie du traitement antiépileptique stable depuis au moins 4 semaines ;<br>- après consentement éclairé du ou des parents ou du représentant légal.
Durée de l'essai	3 ans + 2 ans (MS2) + 1 ans (MS 7)

V- JUSTIFICATION DE L'ETUDE

1.1 Autismes et Troubles Envahissants du Développement (TED)

Les TED sont des troubles graves et chroniques qui entravent les capacités de communication et de socialisation de l'enfant et ils s'accompagnent généralement d'une restriction majeure des intérêts de l'enfant. Dans leur forme typique, les troubles autistiques débutent avant l'âge de 3 ans.

Si de nombreux enfants autistes ou présentant un trouble envahissant du développement non spécifique peuvent bénéficier d'une scolarité en milieu ordinaire, les troubles autistiques s'accompagnent souvent de troubles graves du comportement à type d'instabilité psychomotrice, de stéréotypies envahissantes, d'hétéro ou d'auto-agressivité, voire d'automutilations parfois très graves (allant parfois jusqu'à l'énucléation).

Les enfants souffrants de TED doivent bénéficier le plus précocement possible d'une prise en charge pluridisciplinaire associant des soins intensifs (hospitalisation à temps plein ou en hôpital de jour avec ou sans traitement médicamenteux), une prise en charge éducative/rééducative et une pédagogie adaptée à leur manière concrète de penser. Une prise en charge précoce et intensive est nécessaire en raison de l'importance de la première enfance dans l'établissement des relations interpersonnelles et de l'image du corps. Le dynamisme du développement chez l'enfant et l'adolescent justifie la population de cette étude.

1.2 Traitements psychotropes

Il n'existe à l'heure actuelle aucun médicament spécifique des symptômes centraux des troubles autistiques comme les difficultés d'intégration sociale, les troubles de la communication et du langage ou la restriction des intérêts. En revanche, les traitements de certains symptômes accessibles aux médications (comme les troubles du comportement) ont été très étudiés, quoique rarement au cours d'étude contrôlées en double aveugle contre placebo (Barnard et al. 2003 ; Buitelaar & Willemsen-Swinkels 2000 ; Palermo & Curatolo 2004 ; McDougle et al. 2003 ; Posey & McDougle 2001 ; Tsai 1999).

Le seul médicament à bénéficier de l'Autorisation de Mise sur le Marché pour les troubles du comportement chez l'enfant autiste de plus de 5 ans était la rispéridone (Risperdal®). La rispéridone se situe parmi les médicaments les plus étudiés et les plus prescrits chez les enfants et adolescents souffrant de troubles envahissant du développement. L'efficacité de la rispéridone est démontrée dans les troubles graves du comportement associés aux syndromes autistiques des enfants et adolescents comme l'instabilité psychomotrice, les stéréotypies, l'agressivité et les auto-mutilations (Aman et al. 2005 ; Aman 2005 ; McDougle et al. 2005 ; McDougle et al. 1998 ; Posey & McDougle 2001 ; RUPP 2005 ; RUPP 2002 ; Scahill et al. 2001 ; Shea et al. 2004 ; Troost et al. 2005 ; Williams et al. 2006).

1.3 Packing

Depuis plusieurs années, la technique du packing (ou séances d'enveloppements humides initialement froids) est proposée pour le traitement des enfants et adolescents autistes les plus gravement malades, ou qui présentent des troubles graves du comportement (Delion et coll. 1998). Devant les résultats cliniques positifs obtenus, il semble important aujourd'hui d'en faire une étude systématisée qui permettra d'en valider la pertinence. Aucune donnée scientifique n'est

disponible à l'heure actuelle et il convient donc d'établir scientifiquement la validité de cette méthode de soins. Une recherche comparable ne peut pas être pratiquée sur des personnes majeures en raison de la réactivité à la prise en charge liée au dynamisme psychique important chez les enfants et adolescents en cours de développement.

Malgré la polémique nationale passionnée au sujet du packing (voir à ce sujet les échos synthétiques très objectifs et nuancés parus dans le Lancet [Spinney, 2007]), les rapporteurs du dossier PHRC n'ont soulevé aucune difficulté à sa réalisation d'un point de vue éthique. Les résultats cliniques suggèrent un rapport bénéfice/risque très favorable.

La technique du packing est basée sur réchauffement thermique cutané rapide (de l'ordre de 3 à 5 minutes selon des vérifications par thermomètres cutanés) obtenu en enveloppant le corps de l'enfant dans des serviettes mouillées froides. Ces tissus d'une température proche de 10° en début de séance se réchauffent très rapidement pour avoisiner la température du corps à la fin de la séance. Seule la température superficielle est transitoirement diminuée, le patient n'est jamais placé en hypothermie. Cette technique favorise une utilisation préférentielle de la thermosensibilité sous ses deux voies (thermo-nociceptive quand la température est inférieure à 10° et thermoceptive au dessus). Les séances d'enveloppement ont lieu une ou plusieurs fois par semaines en fonction de l'état de santé du patient. Les packings aident les enfants à renforcer leur conscience des limites de leur corps. Le packing a pour but d'aider le patient à retrouver une image corporelle en privilégiant ses vécus sensoriels et émotionnels. Bien plus qu'une méthode comportementale et uniquement corporelle, le packing permet une contenance psychique de l'enfant qui permet l'établissement d'une relation avec les soignants qui accompagnent attentivement l'enfant pendant les séances.

Les packings entrent dans le cadre d'un projet de soins individualisés en accord avec les parents. Ils n'ont jamais lieu quand ils sont refusés par l'enfant. Cette prise en charge s'intègre au sein d'une prise en charge pluridisciplinaire, qui associe des soins, de l'éducatif et du pédagogique.

Certains résultats (Tordjman & Charras, 2007) suggèrent que l'observation d'une apparente diminution de réactivité à la douleur dans l'autisme infantile ne relèverait pas d'un mécanisme d'analgésie endogène, mais plutôt d'un mode différent d'expression de la douleur, en rapport avec les troubles de la symbolisation, les troubles de la communication verbale et non verbale et certains autres troubles cognitifs : troubles de l'apprentissage et de l'image du corps, problème de représentation des sensations et des émotions, difficultés à établir des relations de cause à effet. Un certain rapport semble exister entre l'amélioration des comportements de réactivité à la douleur et la progression de la construction corporelle telle que mise en évidence par la grille de G. Haag et collaborateurs (2005). Selon Tordjman et Charras, une hypothèse est que certains patients autistes se servent des automutilations pour provoquer une sensation douloureuse, sachant que ces automutilations, d'après les observations cliniques des soignants et des parents, apparaissent essentiellement lorsque les patients sont stressés. Il est possible que cette sensation douloureuse permettrait aux patients de se focaliser complètement sur eux-mêmes, c'est-à-dire de pouvoir accéder à un certain retrait autistique, et de se couper d'un environnement anxiogène comme si cette sensation douloureuse avait un effet hypnotique. Selon ces auteurs, le packing, grâce au saisissement par le froid, mobilise la sensibilité thermo-algique par l'intermédiaire du faisceau spino-thalamique, et court-circuite la sensation douloureuse sur laquelle l'enfant se serait possiblement focalisé. Il s'agit donc par la packing de remplacer une sensation dont le patient est devenu extrêmement dépendant par un autre type de stimulation mobilisant le même circuit neuro-physiologique. Ce « substitut » peut lui permettre de sortir de sa dépendance aux automutilations et des sensations douloureuses qui lui sont associées. Selon Tordjman et Charras, il se peut que la mobilisation des circuits provoquée par le packing soit efficace à long terme contre les comportements d'automutilations. En d'autres termes, si l'enfant avec autisme, focalisé sur la sensation douloureuse qu'il provoque et contrôle à partir de ses automutilations,

concentre maintenant son attention sur le froid engendré par le packing, il est possible que l'on puisse entamer un travail de désaccoutumance par rapport à ses conduites auto-agressives et aux sensations en découlant.

Hypothèse

Les soins par packing peuvent légitimement être proposés aux enfants souffrant de TED les plus gravement malades.
La prise en charge par packing est proposée indépendamment des prescriptions de psychotropes.

VI - OBJECTIF(S) DE L'ETUDE

A) Objectif principal

L'objectif principal est de mesurer l'efficacité à 3 mois des techniques de packing dans les troubles graves du comportement des enfants présentant un TED.

B) Objectifs secondaires

Un objectif secondaire est d'évaluer la place des packings dans la stratégie thérapeutique globale à proposer aux enfants autistes les plus gravement malades.

Un autre objectif est d'évaluer l'importance, éventuellement différente, des éventuelles prescriptions psychotropes dans chacun des groupes (Packings ou enveloppements secs).

C) Critère principal de jugement

Diminution de l'intensité des troubles du comportement objectivés par le sous-score Irritabilité de l'échelle *Aberrant Behavior Checklist* (ABC) à la 12ème semaine (**critère principal**).

D) Critères secondaires de jugement

- CARS entre la randomisation et l'évaluation à 3 mois.
- Différence du score total et des sous-scores d'hyperactivité, de léthargie, et stéréotypie de l'échelle ABC entre la randomisation et l'évaluation à 3 mois.

VII - DESCRIPTION DE L'ETUDE

A) Méthodologie

1) Type d'essai et phase

Essai de phase II.

2) Méthodologie

Essai contrôlé en groupe parallèle avec randomisation. Évaluation en aveugle.

B) Organisation générale de l'étude :

1) Lieux de l'étude

La recherche est réalisée : (voir liste annexe)

- dans les services universitaires de pédopsychiatrie de Lille (Pr Pierre Delion), Caen (Pr Jean-Marc Baleyte) et Amiens (Pr Christian Mille).
- dans les secteurs de pédopsychiatrie de la région Nord – Pas de Calais, qui pratiquent usuellement le packing
- dans certaines structures médico-sociales (Instituts Médico-Educatifs) qui pratiquent usuellement le packing

L'ajout de centres sera sujet à amendements.

- Centre de Santé Mentale angevin CESAM (49) (Dr Charlery Martine)
- Centre hospitalier de Brive (19) (Dr Vaillant Anne Marie)
- Centre hospitalier La Pitié (75) (Dr Xavier Jean)
- Centre de Soins Médico-Psychologique pour enfants
- Service de psychiatrie infanto-juvénile du secteur 49i03 au Centre Hospitalier de Cholet(49) (Dr Stéphanie Dauver)
- Hôpital Maison Blanche - Secteur Buttes Chaumont (75) ; Hôpital de jour « La pomme » (paris 18ème) : Dr Fabienne Roos-Weil
- CH de Lens (62) – Dr Batardière Nathalie
- Hôpital de jour Enfants de Gennevilliers (92) : Dr Louzoune Claude.
- Centre hospitalier de Montfavet (84) : Dr Bonnauron Christine.
- IME la pépinière à Loos (59) : Dr Catot-Larue Claude
- IMA de Montigny en Ostrovent (59) : Dr Lepintre Claude
- EPSM val de lys/artois à St Venant (62) : Dr Gaudry Caroline.
- Centre Hospitalier d'Arras (62)- service pédopsychiatrie 62i07 : Dr Ardouin Geoffrey
- Centre Hospitalier George Mazurelle (85 La Roche sur Yon) : Dr Benbouhou Florence
- Centre Hospitalier de Marne la Vallée - Hôpital de jour l'Olivier (77 Jossigny/ Lagny) : Dr Duguet Marion (MS7)

2) Accueil des sujets et déroulement de l'étude

1. **Recrutement, information des parents, délai de réflexion**

Les patients sont screenés et recrutés sur discussion médicale dans les CMP et services hospitaliers des équipes faisant partie de l'étude. La sélection possible du patient se discute lorsqu'une prise en charge par packing est proposée à un enfant ou un adolescent répondant aux critères d'inclusion de l'étude.

Le médecin investigateur local propose aux parents la participation à cette étude et leur fournit le formulaire d'information et de consentement et les invite à visionner le DVD « Cocon de Soi » réalisé par les parents d'une enfant autiste ayant bénéficié de packings. Les parents bénéficient d'un délai de réflexion d'une semaine (ou plus s'ils le souhaitent).

2. **Visite de pré-inclusion**.

Le médecin investigateur local examine personnellement le patient et procède à la pré-inclusion dans l'étude. Le médecin investigateur local informe les parents sur le déroulement de l'étude et leur fait signer le formulaire d'information et de consentement.
Le médecin organise la première évaluation psychologique de l'enfant.

3. **Visite d'inclusion définitive, Randomisation, 1ère évaluation psychologique**.

Le médecin responsable de la prise en charge du patient réalise l'inclusion dans l'étude avec la participation de l'équipe dédiée du CHU de Lille (MS3)

Un numéro d'anonymisation est attribué par appel téléphonique du médecin investigateur local au CHRU de Lille. La randomisation détermine définitivement le mode de prise en charge : 1) soit bras avec des enveloppements humides (Packings) ; 2) soit bras avec enveloppements secs (couvertures sans linge humide).

Le (la) psychologue réalise auprès de l'enfant, de l'équipe et des parents la première évaluation psychologique (ABC, SIBS, CGI, cahier d'observation) avant tout enveloppement.

4. **Enveloppements secs et humides**

Les enveloppements humides ou secs débutent selon les modalités prévues par le protocole (au rythme de deux séances par semaine). Les enveloppements sont réalisés par deux membres de l'équipe soignante de la structure où le patient est habituellement pris en charge, ou par celle de l'unité d'hospitalisation (à temps plein ou de jour) où l'enfant est hospitalisé. En fonction des besoins, cette équipe est éventuellement aidée ou assistée par le personnel infirmier recruté par le financement PHRC. Les personnels seront formés à la technique du packing par l'équipe du service de psychiatrie de l'enfant et de l'adolescent du Pr Pierre Delion, CHU de Lille. Les collaborations sanitaires et médico-sociales sont possibles.

Le (la) psychologue réalise en aveugle (c'est-à-dire en ignorant le mode de prise en charge) sur le lieu de prise en charge, auprès de l'enfant, de l'équipe et des parents, les évaluations psychologiques (ABC, CARS, SIBS, CGI, cahier d'observation) au tout début de l'inclusion (avant le début des enveloppements), puis après 1, 2 et 3 mois.

5. **Suivi de l'étude à 1, 2 et 3 mois**

A chaque date de suivi mensuel, le (la) psychologue réalise les évaluations psychologiques.
Le médecin investigateur local veille au bon déroulement de l'étude et consigne mensuellement sur un carnet de bord (calendrier de traitement) les évolutions cliniques qui seront reprises par l'ARC sur le cahier d'observation.

À l'issue des trois mois de l'étude, le cahier d'observation est adressé à l'investigateur principal, au CHRU de Lille. Les données seront ensuite saisies par un(e) attaché(e) de recherche clinique financée par le PHRC.

6. **Fin de l'étude**.

À l'issue des trois mois de prise en charge, l'enfant sort du protocole d'étude, la poursuite des soins étant sous la responsabilité du médecin responsable localement de sa prise en charge habituelle.

a. Dans le cas où les enveloppements secs s'avèrent inefficaces, leur arrêt est possible (après les 3 mois protocolaires). Dans ce cas, la réalisation de packings humides et initialement froids est possible en dehors du cadre de l'étude sur décision du médecin responsable des soins de l'enfant.
b. Dans le cas où les packings ou les enveloppements secs ont été efficaces, leur poursuite est bien entendu toujours possible selon l'avis du médecin responsable.

Définitions

Packing : enveloppements dans des serviettes de bain humides et initialement froides (10-15°C environ). Les serviettes sont recouvertes d'une alaise et de couvertures pour faciliter le réchauffement de l'enfant (en 3 à 5 minutes en pratique). Les séances durent 45 minutes environ. Le rythme est de deux séances par semaine pendant les 3 mois de l'étude. Le nombre d'enveloppement (humide ou sec) par semaine doit être respecté ensuite. Le sujet est constamment accompagné par les personnes formées qui réalisent l'enveloppement. À l'issue de la séance, le sujet est frictionné et accompagné vers le lieu de vie où une collation peut être proposée dans une ambiance conviviale.

Enveloppements secs : enveloppements dans une couverture simple dans les mêmes conditions de placement du corps, de rythme et de personnel mobilisé qu'au cours des séances de packing.

Remarque : Les enveloppements limitent de manière souple la mobilité de l'enfant. Celui-ci ne peut pas se dégager des linges, mais il peut bouger et se redresser. Il ne s'agit pas d'une contention avec entraves sur un lit. Seuls les membres supérieurs et inférieurs et le tronc sont enveloppés. La tête et le visage ne sont pas concernés.

VIII - DESCRIPTION DES SUJETS

L'étude s'adresse à des sujets volontaires malades, mineurs et incapables de donner leur consentement (sans langage ou trop déficients).

A) Calcul du nombre de sujets nécessaires

Il s'agit d'une étude pilote. Nous proposons de recruter 20 sujets par groupe afin d'estimer avec une précision suffisante la taille de l'effet (effect size). La taille de l'effet est égale à la différence des moyennes observées entre les groupes, divisée par la déviation standard estimée. Elle permet donc de normaliser la différence observée par rapport à la déviation standard.

D'après Cohen (Cohen J, A power primer, Psychological Bulletin 112 (1): 155–159, 1992), la taille de l'effet s'interprète de la manière suivante : 0,2 correspond à une taille de l'effet faible, 0,5 à une taille de l'effet moyenne et 0,8 à une taille de l'effet importante. L'estimation de la taille de l'effet pour cette étude sera interprétée selon ces règles. Cette étude devrait permettre de déterminer si la taille de l'effet est suffisante.

B) Critères d'inclusion

Il n'y a pas lieu d'exclure les enfants présentant des anomalies génétiques ou une pathologie neurologique particulière. Dans le cas particulier de l'épilepsie, le traitement devra être équilibré (Cf. protocole).

Les Quotients Intellectuels (WISC, K-ABC) et les Quotients de Développement (Brunet-Lezine) et le Profil Psycho-Educatif (PEP-R) n'interviennent pas dans les critères d'inclusion, mais leur renseignement est utile (chez les enfants et adolescents qui peuvent être testés malgré leurs déficiences éventuelles ou leurs troubles graves du comportement) pour la description de la population étudiée, notamment afin d'évaluer l'efficacité éventuellement différente des packings selon le QI/QD de l'enfant.

Un bilan neuropédiatrique fait partie de l'évaluation clinique habituelle des enfants et adolescents autistes. Sa réalisation peut avoir lieu au cours ou après les enveloppements. Les examens complémentaires éventuels (EEG, Scanner, IRM, caryotype, X-fragile, recherche du gène MECP-2, etc) sont prescrits en dehors du cadre de l'étude.

Critères :

- Enfants et adolescent(e)s âgés de plus de 3 ans(MS 3) présentant selon les critères internationaux (CIM 10) évalués par l'ADI-R : un syndrome autistique, un syndrome d'Asperger, ou un trouble envahissant du développement non spécifique ;
- présentant des troubles graves du comportement (tels que auto- ou hétéro-agressivité, auto-mutilations, instabilité psychomotrice sévère, stéréotypies graves et envahissantes) objectivés par les scores : sous-score Irritabilité de l'échelle ABC>18, CGI>4 ;
- ayant bénéficié d'une consultation neuropédiatrique (qui déterminera de l'opportunité de réaliser des examen complémentaires) ;
- pour les patients épileptiques : posologie du traitement antiépileptique stable depuis au moins 4 semaines ;
- après consentement éclairé du ou des parents ou du représentant légal.

C) Critères de non-inclusion

- Sujets présentant un syndrome de Rett ou un trouble désintégratif de l'enfance ;
- sujets présentant des antécédents de syndrome malin aux neuroleptiques ;
- absence d'accord parental après information ;

D) Bénéfices attendus pour le sujet

Diminution des troubles graves du comportement, des stéréotypies, de l'hyperactivité, des insomnies, des comportements d'auto- ou d'hétéro-agressivité.

Meilleure prise de conscience de l'image corporelle.

Amélioration du contact relationnel avec autrui, amélioration de la qualité de vie (diminution de la fréquence et des temps de contention)

E) Critères d'arrêt de l'étude pour un sujet qui y participe

Chaque sujet pourra sortir de l'étude en cas d'impossibilité de réalisation des enveloppements, de refus manifeste de sa part, ou de refus de l'un de ses parents. Les données déjà recueillies sont prises en compte dans la description finale de l'étude.

Chaque sujet pourra sortir de l'étude par décision de l'autorité administrative compétente, du promoteur et de l'investigateur coordonnateur mais aussi par décision d'un co-investigateur ou par décision de l'intéressé lui-même conformément à la réglementation et comme il est mentionné dans le consentement.

IX – TRAITEMENTS

L'inclusion dans l'étude n'est subordonnée à aucune prescription.

Traitements concomitants

Avant et/ou pendant l'essai, sont

- autorisés : tous les traitements. Toute modification du traitement médicamenteux (ajout retrait d'un médicament, augmentation ou diminution de posologie) au cours de l'expérimentation devra faire l'objet d'une justification.
- interdits : tout traitement expérimental.

La prescription éventuelle est réalisée sous la responsabilité du médecin responsable de la prise en charge globale de l'enfant, en aucun cas elle ne peut être motivée par l'inclusion dans le protocole.

X – BIOLOGIE

Aucun examen supplémentaire aux pratiques habituelles n'est demandé dans le cadre de l'étude : les évaluations cliniques et biologiques réalisées au cours de cette étude correspondront aux recommandations d'évaluations au cours de la prise en charge d'un enfant ou d'un adolescent souffrant d'un Trouble Envahissant du Développement, et aux recommandations de surveillances médicales d'un traitement antipsychotique, notamment :

Une mesure du poids et de la taille au début et à la fin de l'étude est importante, car l'indice de corpulence (BMI=P/T²) ajusté au sexe et à l'âge de l'enfant est un moyen commode et fiable de surveillance d'une éventuelle prise de poids, notamment dans les cas où une prescription de psychotropes est nécessaire.

D'autres mesures sont utiles : Pression Artérielle, Tour de Taille, Tour de Hanche, Glycémie à jeun, Cholestérol Total et Triglycérides, mais aucune prise de sang n'est nécessaire pour l'inclusion dans l'étude.

De même, aucun enregistrement ECG n'est nécessaire pour l'inclusion dans l'étude.

XI - EVENEMENTS INDESIRABLES GRAVES

A) Définitions

Événement indésirable

Sont considérés comme **événements indésirables**, toute manifestation nocive survenant chez une personne qui se prête à une recherche biomédicale, que cette manifestation soit liée ou non à la recherche ou au produit sur lequel porte cette recherche.

Événement indésirable grave

Sont considérés comme **événements indésirables graves** : les décès, et tous les événements qui surviennent durant l'étude et qui :
- mettent en jeu le pronostic vital,
- nécessitent un prolongement d'hospitalisation,
- entraînent des lésions ou des séquelles irréversibles,
- sont jugés comme graves par l'investigateur.

Événement indésirable inattendu

Un événement indésirable doit être considéré comme inattendu si sa nature, son intensité ou son évolution ne concordent pas avec les informations contenues dans le protocole.

B) Événements indésirables et risques prévisibles liés au protocole

Les risques prévisibles liés au protocole sont :

- risques liés à la technique de packing :

Chute sur le sol de la hauteur du lit où l'enfant est allongé pour être enveloppé. En cas de difficulté, les enveloppements peuvent être réalisés avec le matelas à même le sol.

Comité de surveillance :

Il a une fonction consultative lorsque le promoteur fait appel à lui sur des points médicaux tels la tolérance et évènements indésirables. Il est constitué de personnes extérieures à l'étude dont un clinicien spécialiste de la pathologie étudiée (Monsieur le Pr Claude Bursztejn, CHU de Strasbourg), un membre d'association de parents d'enfants autistes (Mme Ghislaine Meillier, ancienne Présidente de l'Association Sésame Autisme Nord) et un pharmacologue (Le Pr Régis Bordet, Institut de Médecine Prédictive et de Recherche Thérapeutique, INSERM, IFR 114).

C) Déclaration

L'investigateur doit notifier au promoteur, sans délai à compter du jour où il en a connaissance, tous les événements indésirables graves.

Tous les événements indésirables graves devront faire l'objet d'un rapport sur un formulaire « Événement Indésirable Grave » figurant dans le cahier d'observation. Ce formulaire devra être transmis au promoteur (Cellule Vigilance de la Fédération de Recherche Clinique) par fax au 03 20 44 57 11.

Le suivi des événements indésirables sera assuré par l'investigateur jusqu'à disparition du symptôme.

La relation de causalité entre l'événement et les produits de l'étude peut être de plusieurs ordres :

- sans relation de causalité avec la recherche biomédicale,
- avec relation de causalité :
  - douteuse
  - possible
  - probable

Tous les événements jugés, soit par l'investigateur, soit par le promoteur, comme n'étant pas «sans relation de causalité avec la recherche biomédicale» sont des suspicions d'événements indésirables.

Le promoteur a la responsabilité de déclarer à la DGS et au CPP, les événements indésirables graves et inattendus et imputables aux procédures de l'étude et/ou aux médicaments utilisés dans un délai de 7 jours.

XII - METHODES D'ANALYSE DES PARAMETRES MESURES

Plan d'analyse statistique détaillée :

Les analyses statistiques seront réalisées à l'aide du logiciel SAS (version 9.3 ou supérieure) et conduite à la plateforme d'aide méthodologique du CHRU de Lille sous la responsabilité du Pr A. Duhamel. Tous les tests statistiques seront bilatéraux avec un risque de première espèce de 5%. Les variables quantitatives seront décrites par la moyenne et l'écart type en cas de distribution gaussienne, ou par la médiane et l'interquartile (i.e. 25ième et 75ième percentiles) dans le cas contraire. La normalité des distributions sera testée par un test de Shapiro-Wilk et vérifiée graphiquement par des histogrammes. Les variables qualitatives seront décrites par les effectifs et pourcentages de chaque modalité.

Echantillon d'analyse :

Etant donné qu'il s'agit d'un essai de Phase II dont l'objectif principal est l'estimation de l'effet de la technique Packing, toutes les analyses seront effectuées sur l'échantillon Per-Protocol (PP). La population PP est définie par les patients randomisés sans déviation majeure au protocole. Les déviations majeures sont les suivantes : les inclusions à tort, les patients non-traités (sans séance d'enveloppement quelques soit le bras de l'étude), et les patients pour lesquels on ne dispose pas du critère principal, incluant les sorties d'études. Le nombre total de patients randomisés, le nombre de patients inclus dans l'analyse per-protocole et le détail des déviations majeurs seront décrit sous la forme d'un flow-chart.

Comparabilité des groupes :

Les caractéristiques de bases (avant traitement) seront décrites et comparées entre les deux groupes afin de s'assurer de la comparabilité des groupes. Les variables quantitatives seront comparées par un test T de Student ou par un test U de Mann-Whitney en cas d'écart à la normalité. Les variables qualitatives seront comparées entre les groupes par un test du Chi-deux ou par un test exact de Fisher (lorsque les conditions de validité du test du Chi-deux ne sont pas vérifiées).

Analyse du critère principal :

Le critère d'évaluation principal (changement du score d'irritabilité de l'échelle ABC) sera comparé entre les deux groupes par une analyse de covariance ajustée sur le score d'irritabilité à l'entrée (avant traitement). Nous estimerons à partir de ce modèle, la différence des moyennes de changement du score (expérimental vs. control) par intervalle de confiance à 95%. La taille de l'effet associée à l'utilisation du Packing sera également calculée (différence de moyenne standardisée, « indice d de Cohen ») ; l'intervalle de confiance à 95% de la taille de l'effet sera calculé par bootstrap (re-échantillonnage). Un diagnostic de validité du modèle sera réalisé (normalité des résidus, valeurs influentes). En cas d'écart à la normalité des résidus, la différence relative du score d'irritabilité entre la randomisation et 3 mois sera calculée et comparée entre les deux groupes par un test U de Mann-Whitney.

Analyse des critères secondaires :

La fréquence des patients avec une amélioration nette de l'impression clinique globale sera comparée entre les deux groupes par un test du Chi-deux ou par un test exact de Fisher. La

taille de l'effet sera calculée par la différence de fréquence absolue et relative (expérimental vs. contrôle).

La même stratégie d'analyse sera utilisée pour comparer la fréquence de patients avec une amélioration très nette de l'impression clinique globale entre les deux groupes.

La stratégie d'analyse décrite pour l'analyse du critère principal sera utilisée pour comparer le changement du score total de l'échelle ABC, des sous-scores d'hyperactivité, de léthargie, et stéréotypie de l'échelle ABC et du score CARS entre les deux groupes.

Analyses Exploratoires :

Une première analyse exploratoire sera réalisée sur l'ensemble des mesures répétées du score total et des sous-scores ABC (M0, M1, M2, M3). Les mesures répétées seront comparées entre les deux groupes par un modèle linéaire mixte. Ce modèle est extension de l'analyse de la variance classique qui permet de prendre en compte la corrélation entre deux mesures successives d'un patient. Le choix de la structure de covariance sera basé sur le critère d'AIC.

Une seconde analyse exploratoire sera réalisée pour étudier la liaison entre l'âge et le critère principal d'une part, et entre le nombre de séance d'enveloppement et le critère principal d'autre part. Le coefficient de corrélation de Spearman sera calculé dans chaque groupe séparément.

Une troisième analyse exploratoire sera réalisée en ajustant la comparaison du critère principal sur l'âge des patients, en incluant l'âge dans le modèle d'analyse de covariance. Si l'analyse principale est effectuée par le test U de Mann-Whitney, un modèle de covariance sur les rangs des données (non-paramétrique) sera utilisé.

XIII - DUREE DE L'ETUDE

- Durée prévue pour le recrutement : 3 ans + 2 ans **+ 1 an (MS7)**
- Durée de l'étude, de la première inclusion à la dernière visite du dernier patient : 6 ans et 3 mois
- Durée de la participation prévue pour un sujet donné : (préparation, traitement : entre 1 et 3 mois, suivi : 3 mois)
- L'essai se terminera lorsque lorsque plus de 40 sujets auront été inclus, mais ne devra pas dépasser 72 mois au total. (jusqu'en décembre 2014).

Le promoteur se réserve le droit d'interrompre l'essai en raison d'un défaut d'inclusions.

Il n'y a pas lieu d'interdire l'inclusion des enfants et adolescents inclus dans ce protocole dans d'autres protocoles que ceux visant à évaluer l'impact de prises en charge des enfants et adolescents souffrants de TED avec troubles graves du comportement

L'étude pourra être arrêtée par décision de l'autorité compétente, du promoteur ou de l'investigateur coordonnateur.

XIV - RESPONSABILITES (LEGALES ET PRATIQUES)

A) Moyens nécessaires

1) *Personnel nécessaire*

Les enveloppements seront réalisés par au moins 2 personnels (infirmièr(e)s diplomé(e)s d'État ou psychomotricien(ne)s ou éducateurs) qui accompagneront le sujet au cours des séances.

Les évaluations psychologiques seront réalisées à l'inclusion, puis à M1, M2, M3 et M4 par un(e) psychologue clinicien(ne).

Les données seront saisies par un(e) attaché(e) de recherche clinique (25% ETP par an pour l'ensemble des centres d'évaluation).

2*) Moyens financiers*

Le financement par le PHRC 2007/1918 permet l'embauche et les déplacements de 3 IDE à temps plein (6 IDE mi-temps), de trois mi-temps de psychologue et d'un mi-temps d'ARC.

B) Type de participation attendu des différents acteurs

	Investigateur principal	*Investigateurs*	*Promoteur (DRC)*	*Autre, préciser :service de soins*	*Charge de travail (en nombre d'heures)*
**Accompagnement méthodologique (protocole CRF)**	x		x		
**CV datés et signés**	x	x			
**Soumission CPP et Ministère**			x		
**Assurance**			x		
**Déclarations administratives**			x		
**Convention financière**			x		
**Elaboration du CRF**	x		x		
**Mise en place**	x		x		
**Stockage des traitements**				x	
**Recrutement des patients**	x	x			
**Recueil du consentement/Information du patient**	x	x			
**Randomisation**	x	x	x		
**EIG**	x		x		
**Pharmacovigilance**			x		
**Monitoring/Suivi de l'état d'avancement**			x		
**Recueil de données/remplissage des CRF**	x	x		x	
**Stockage prélèvements/Traçabilité**	x	x		x	
**Amendements**			x		
**Fermeture du (ou des) centre(s)**	x		x		
**Analyse des données**	x			x	
**Clôture**			x		
**Rapport final**	x				
**Archivage**			x		

C) Soumission pour avis au CPP et pour autorisation à la DGS

Le promoteur a soumis le protocole pour avis au CPP Nord Ouest et pour autorisation à la DGS conformément à la loi N° 2004-806 du 9 août 2004 relative à la politique de santé publique et son décret d'application n°2006-477 du 26 avril 2006 ainsi que les arrêtés et décisions s'y rapportant.

D) Amendements au protocole

Il n'y aura pas d'altération ou de changement à ce protocole sans l'accord de l'ensemble des investigateurs et du promoteur.

Conformément aux dispositions de l'article R 1123-35 du code la santé publique, les modifications substantielles sont celles qui ont un impact significatif sur tout aspect de la recherche notamment sur les critères suivants :

- la protection de personnes, y compris à l'égard de leur sécurité
- les conditions de validité de la recherche
- le cas échéant, la qualité et la sécurité des produits expérimentés (médicaments expérimentaux et éventuels autres produits utilisés dans le cadre de la recherche)
- l'interprétation des documents scientifiques qui viennent appuyer le déroulement de la recherche
- les modalités de conduite de la recherche

Seules les modifications substantielles sont soumises à autorisation et/ou avis de la DGS et du CPP. Les modifications non substantielles sont transmises au CPP pour information.

E) Information des sujets et recueil écrit du consentement éclairé

Une information complète et loyale (en des termes aisément compréhensibles par le sujet) doit être fournie et une lettre d'information (cf. annexe) doit être remise au sujet par l'investigateur ou son représentant **déclaré**.

Elle précise les objectifs, les méthodes et la durée de la participation (pour un sujet donné), les principales contraintes du protocole, les risques éventuels encourus et les mesures de sécurité prises. Elle rappelle que le sujet peut refuser, et à tout moment et sans préjudice personnel, retirer son consentement et qu'il peut être informé des résultats globaux de l'étude lorsque celle-ci est terminée.

Un formulaire de consentement éclairé (cf. annexe) sera également rédigé.

L'adhésion du sujet à la recherche ne sera pas recherchée. Une information sera réalisée à l'aide par exemple d'une poupée enveloppée de bandes de tissu et du visionnage du DVD « Un cocon de Soi » réalisé par les parents d'une enfant autiste ayant bénéficié de packings.

Le formulaire de recueil de consentement est signé, selon les cas :
- par les deux titulaires de l'autorité parentale (parents),
ou
- par le représentant légal du mineur, ou le conseil de famille ou le juge des tutelles, si le mineur est sous tutelle.

La lettre d'information et le formulaire de recueil de consentement éclairé sont signés par les parents de l'enfant ou tout autre personne autorisant la participation à la recherche (ex : représentant légal, juge des tutelles), ainsi que par l'investigateur déclaré.

Un exemplaire de ces documents sera remis aux personnes autorisant la participation de l'enfant à la recherche ; un autre sera conservé par l'investigateur.

Seul un investigateur **déclaré** peut signer ces documents.

F) Cahiers d'observation

Un cahier d'observation doit être rempli pour chaque patient inclus dans l'étude. Ce cahier sera anonymisé pour assurer la confidentialité des données.

Durant l'étude, les cahiers d'observation seront conservés dans le bureau de l'investigateur et seront remplis par ce dernier.

A la fin de l'étude une fois la clôture faite, les cahiers d'observation seront regroupés et conservés au CHU de Lille.

G) Assurance qualité

L'étude sera suivie par un attaché de recherche clinique qui, à des intervalles réguliers s'assurera que l'étude se déroule conformément au protocole, aux Bonnes Pratiques Cliniques et à la réglementation en vigueur.

Il sera responsable de la vérification des cahiers d'observation afin de s'assurer qu'ils sont complètement et clairement remplis et que les données sont conformes aux documents sources.
L'investigateur assure le promoteur de sa coopération dans cette vérification.
Par ailleurs, l'investigateur comme le promoteur pourront faire l'objet d'une inspection par les autorités compétentes.

Un monitoring de l'étude sera réalisé selon le modèle du risque B c'est à dire risque prévisible proche de celui des soins usuels (concerne les essais cliniques de recherches thérapeutiques sur l'utilisation de techniques standardisées, mais dont l'efficience n'est pas démontrée).

Le monitoring portera sur :
- l'existence des patients inclus
- les consentements éclairés signés
- les critères d'éligibilité
- le critère de jugement principal
- les évènements indésirables et EIG
- la gestion des produits à l'étude.

XV - CONFIDENTIALITE

Les données seront anonymes et répertoriées selon un numéro de région, de centre et de patient.

L'investigateur conservera pour son usage une liste des sujets associant les identités, adresses, numéros de téléphone et numéros de dossier, aux numéros, initiales ou codes sous lesquels ils apparaissent sur les documents relatifs à l'essai. Cette liste sera archivée avec les documents relatifs à l'essai.

Les données et prélèvements seront traités de façon confidentielle (secret médical) et il ne sera en aucun cas possible au promoteur de connaître ou deviner l'identité d'un sujet. Le traitement des données sera réalisé dans les conditions de confidentialité définies par la relative à l'informatique, aux fichiers et aux libertés, modifiée par la loi N° 2004-801 du 6 août 2004 et des textes réglementaires pris pour son application.

XVI - ASSURANCE

Le CHRU en tant que Promoteur aura souscrit un contrat d'assurance "Adhésion au Contrat Promoteur de Recherche Biomédicale" (SHAM, Lyon France), garantissant, dans ses conditions et limites, la responsabilité civile légale pouvant incomber à l'investigateur et à ses collaborateurs en raison des dommages causés aux sujets et résultant de la présente recherche effectuée conformément aux instructions du protocole et dans le respect de la réglementation et de usages professionnels en vigueur.

XVII- CONVENTIONS FINANCIERES

Le budget de l'étude est sous la responsabilité du promoteur qui doit s'assurer que celui-ci est suffisant pour le bon déroulement de l'étude.
Le projet a reçu un financement dans le cadre du PHRC 2007.

XVIII - RAPPORT FINAL ET PUBLICATIONS

Les résultats de l'étude seront proposés à la publication à la fin de l'étude dans des revues internationales de psychiatrie. Le Dr Jean-Louis Goëb, et Pierre Delion seront co-auteurs, après analyse des données. Mention sera faite dans ces publications du rôle de promoteur du CHRU de Lille.

Le rapport final de l'étude sera rédigé par le Dr Jean-Louis Goëb, et le Pr Pierre Delion.

Si d'éventuels brevets, directement issus de cette étude, étaient déposés, cela se ferait après concertation avec le promoteur de l'étude, et les retombées scientifiques et financières seraient réparties après entente préalable entre les différents partenaires (CHRU de Lille, INSERM, Université de Lille 2 et Institut Pasteur de Lille) au prorata de leur travail effectif et de leur participation financière aux dépôts des brevets.

XIX - RESPONSABILITES DE L'INVESTIGATEUR

Chaque investigateur s'engage à respecter scrupuleusement le protocole et les règles de Bonnes Pratiques Cliniques (BPC).

Il s'engage à accepter les contrôles d'un ARC et à fournir l'accès aux données sources *(dossiers médicaux, fichiers informatiques, documents de l'étude…).*
Lors de ces visites sur site en accord avec les BPC, les éléments suivants sont revus :
- respect du protocole et des procédures qui y sont rattachées
- assurance qualité des données recueillies dans le cahier d'observation : exactitude, données manquantes, cohérence des données, contrôle des documents sources.

L'investigateur s'engage à garder des données sources ainsi que les documents administratifs relatifs au protocole et à ne pas inclure de volontaire avant la réception des avis officiels du CPP et de l'envoi de la déclaration aux autorités, à respecter le protocole, à conduire l'étude suivant les principes moraux, réglementaires, éthiques et scientifiques qui régissent la recherche clinique. Il s'engage également à recueillir le consentement éclairé et écrit de chaque volontaire et à rapporter tout événement indésirable grave.

Il inscrit la recherche dans une base de donnée européenne.

Durée de l'archivage des données de l'étude et des consentements : 15 ans.

XX - ANNEXES

- Lettre d'information et formulaire de consentement éclairé (cf annexes 1 et 2)

- budget de l'étude et conventions financières (cf annexe 3)

- CV abrégé des investigateurs avec leurs publications (cf annexe 4).

REFERENCES BIBLIOGRAPHIQUES

Aman MG, Arnold E, McDougle CJ, et al. Acute and long-term safety and tolerability of risperidone in children with autism. J Child Adolesc Psychopharmacology 2005 ; 15 : 869-884.

Aman MG, Singh NN, Stewart AW, et al. The Aberrant Behavior Checklist : A behavior rating scale for the assessment of treatment effects. Am J Ment Deficiency 1985 ; 89 : 485-491.

Aman MG. Treatment planning for patients with autism spectrum disorders. J Clin Psychiatry 2005; 66 Suppl 10 : 38-45.

Arnold LE, Aman Mg, Martin A, et al. Assessment in multisite randomised clinical trials of patients with autistic disorders: The autism RUPP Network. J Autism Dev Disord 2000 ;30 : 99-111.

Barnard L, Young AH, Pearson J, et al. A systematic review of the use of atypical antipsychotics in autism. J Psychopharmacol 2003 ;16 : 93-101.

Buitelaar JK, Willemsen-Swinkels SHN. Medication treatment in subjects with autistic spectrum disorders. Eur Child adolesc Psychiatry 2000; 9 : I/85-I/97.

Carter AS, Volkmar FR, Sparrow SS, et al. The Vineland Adaptative Behavior Scale : Supplementary norms of individuals with autism. J Autism Dev Disord 1998; 28: 287-302.

Correll CU, Carlson HE. Endocrine and metabolic Adverse effects of psychotropic medications in children and adolescents. J Am Acad Child Adolesc Psychiatry 2006; 45: 771-91.

Correll CU, Penzer JB, Parikh UH, et al. Recognizing and monitoring adverse events of second-generation antipsychotics in children and adolescents. Clin Adolesc Psychiatric N Am 2006;15: 177-206.

Correll CU. Metabolic Side effects of second-generation antipsychotics in children and adolescents : A different story ? J Clin Psychiatry 2005 ; 66 : 1331-2.

Delion P. Le packing avec les enfants autistes et psychotiques. 1998 : Erès ; Toulouse.

Diagnostic and statistical manual of mental disorders, 4th ed. DSM-IV. Washington, DC : American Psychiatric Association ; 1994.

Fenton WS, Chavez MR. Medication-induced weight gain and dyslipidemia in patients with schizophrenia. Am J Psychiatry 2006 ; 163 : 1697-1704.

Haag G, Tordjman S, et al. Grille de repérage clinique des étapes évolutives de l'autisme infantile traité. Psychiatrie de l'enfant 1995 ; 38 : 495-527.

Lord C, Pickles A, McLennan J, et al. Diagnosing autism : analyses of data from the Autism Diagnostic Interview. J Autism Dev Disord 1997 ; 27 : 501-17.

Lord C, Rutter M, Le Couteur AM. Autisme Diagnostic Interview-Revised : A revised version of a diagnostic interview for caregivers of individuals with possible pervasive developmental disorders. J Autism Dev Disord 1994 ; 24 : 659-85.

McDougle CJ, Holmes JP, Carlson DC, et al. A double-blind, placebo-controlled study in adults with autistic disorder and other pervasive developmental disorders. Arch Gen Psychiatry 1998 ; 55 :633-41.

McDougle CJ, Scahill L, Aman MG, et al. Risperidone for the core symptom domains of autism : Results from the study by the Autism Network of the Research Units on Pediatric Psychopharmacology. Am J Psychiatry 2005 ; 162 : 1142-8.

McDougle CJ, Stigler KA, Posey DJ. Treatment of aggression in children and adolescents with autism and conduct disorders. J Clin Psychiatry 2003 ; 64 suppl 4 : 16-25.

Palermo MT, Curatolo P. Pharmacologic treatment of autism. J clin Neurol 2004 ; 19 : 155-164.

Posey DJ, McDougle CJ. Pharmacotherapeutic management of autism. Exp Opin Pharmacother. 2001 ; 2 : 587-600.

Psychopharmacology Bulletin. Special features : Rating scales and assessment instruments for use in pediatric psychopharmacology research. Psychopharm Bull 1985 ; 21 : 713-111.

Research Units on Pediatric Psychopharmacology Autism Network. Risperidone treatment of autistic disorder : Longer-term benefits and blinded discontinuation after 6 months. Am J Psychiatry 2005 ; 162 : 1361-9.

Research Units on Pediatric Psychopharmacology Autism Network. Risperidone in children with autism and serious behavioral problems. N Engl J Med 2002 ; 347 : 314-21.

Scahill L, McCraken J, McDougle CJ, et al. Methodological issues in designing a multisite trial of risperidone in children and adolescents with autism. J Child Adolesc Psychopharmacology 2001 ; 11 : 377-88.

Spinney L. Therapy for autistic children causes outcry in France. Lancet 2007 ;370 :645-646, 25 août 2007.

Schopler E, Reichler RJ, De Vellis RF, et al. Toward objective classification of childhood autism : Childhood Autism Rating Scale (CARS). J Autism Dev Disord 1980 ; 10 :91-103.
Shea S, Turgay A, Caroll A, et al. Risperidone in the treatment of disruptive behavioral symptoms in children with autistic and other pervasive developmental disorders. Pediatrics 2004 ; 114 : 634-41.

Tordjman S, Charras K. Intérêts d'une meilleure compréhension de l'apparente insensibilité à la douleur et des automutilations dans l'autisme : vers de nouvelles perspectives thérapeutiques. In: Delion P (Ed) La pratique du packing avec les enfants autistes et psychotiques en pédopsychiatrie. Ramonville: Eres; 2007. Pp 49-61.

Troost PW, Lahuis BE, Steenhuis MP, et al. Long-term effects of risperidone in children with autism spectrum disorders : A placebo Discontinuation study. J Am Acad Child Adolesc Psychiatry 2005 ; 44 : 1137-44.

Tsai LY. Psychopharmacology in autism. Psychosomatic Medicine 1999 ; 61 : 651-665.

Vieweg WVR, Sood AB, Pandurangi A, et al. Newer antipsychotic drugs and obesity in children and adolescents. How should we assess drug-associated weight gain ? Acta Psychiatr Scand 2005 111 :177-84.

William SK, Scahill L, Vitiello B, et al. Risperidone and adaptative behavior in children with autism. J Am Acad Child Adolesc Psychiatry 2006 ; 45 : 431-9.
