# Supplementary material for: Therapeutic body wraps (TBW) for treatment of severe injurious behaviour in children with autism spectrum disorder (ASD): A 3-month randomized controlled feasibility study
Source: PLoS One. 2018 Jun 29;13(6):e0198726. doi: 10.1371/journal.pone.0198726 (PMC6025870; doi:10.1371/journal.pone.0198726)
Supplement: S9 File — (PDF) [file pone.0198726.s009.pdf]

DEMONSTRATION OF THE EFFECTIVENESS OF THERAPEUTIC BODY WRAP IN CHILDREN SUFFERING FROM AUTISM DISORDERS WITH SERIOUS BEHAVIORAL DISORDERS

Principal investigator:

Pr Pierre Delion
Service de Psychiatrie de l'Enfant et de l'Adolescent
du Pr Pierre DELION,
Hôpital Michel Fontan,
Centre Hospitalier Universitaire de Lille

Co-investigators

Pr Jean-Marc BALEYTE,
Service de Psychiatrie de l'Enfant et de l'Adolescent,
Centre Hospitalier Universitaire de Caen

Pr Christian MILLE,
Service de Psychiatrie de l'Enfant et de l'Adolescent,
Centre Hospitalier Universitaire d'Amiens

Other Investigateurs

CHU de LILLE : Dr Jean-Louis GOEB, Dr Anne-Yvonne LENFANT, Dr Renaud JARDRI, Dr Frank BONELLI, Dr Géraldine KECHID.

Study sponsor

CHRU de Lille

2, avenue Oscar Lambret

59037 Lille Cedex

II – DATES and SIGNATURES

Identity	Date	Signature
Dr Jean Louis GOEB		
Pr DELION		
Dr Anne-Yvonne LENFANT,		
Dr Renaud JARDRI		
Dr Frank BONELLI		
Dr Géraldine KECHID		
Pr Christian MILLE		
Pr Jean Marc BALEYTE		

III - SUMMARY

I TITLE PAGE *1*
II DATES AND SIGNATURES *2*
III SUMMARY *3*
IV SYNOPSIS OF THE PROJECT *5*
V STUDY JUSTIFICATION *6*
1.1 Autism and Pervasive developmental disorder (PDD) 6
1.2 Psychotropic treatments 6
1.3 Therapeutic body wrap 6
VI STUDY AIMS 8
A) Primary objective 8
B) Secondary objectives 8
C) Primary variable 8
D) Secondary variables 8
VII STUDY DESCRIPTION *9*
A) Methodology 9
B) Study general organisation 9
VIII PARTICIPANTS' DESCRIPTION *12*
A) Sample size calculation 12
B) Inclusion criteria 12
C) Non inclusion criteria 13
D) Expected benefits for the participant 13
E) Criteria for participant's withdrawal from the study 13
IX TREATMENTS *13*
X BIOLOGY 13
XI SERIOUS ADVERSE EVENTS *14*
A) Definition 14
B) Adverse events and foreseeable risks related to the protocol 14
C) Declaration 15
XII STATISTICAL ANALYSIS PLAN 15
XIII STUDY DURATION *16*
XIV RESPONSABILITIES (LEGAL AND PRATICAL) *16*
A) Necessary means 16
B) Type of participation expected for the different actors 17
C) Submission to the CPP for opinion and to the DGS for authorization 18
D) Amendments to the protocol 18
E) Subject Information, Written and Informed Consent 18
F) Protocol observation books 19
G) Quality assurance 19
XV CONFIDENTIALITY **20**

XVI INSURANCE **20**

XVII FINANCIAL CONVENTIONS **20**

XVIII FINAL REPORT AND PUBLICATIONS **20**

XIX INVESTIGATOR'S RESPONSABILITIES **21**

XX ANNEXES **21**

IV – SYNOPSIS

SYNOPSIS

Sponsor	CHRU de LILLE
Justification	Some children and adolescents with autistic syndrome have serious behavioral problems that are poorly controlled by their usual management. The care by therapeutic body wrap (TBW) gives valuable clinical results that should be established scientifically to clarify the place of this care in the context of a comprehensive care of the child (therapeutic, educational and educational).
Aims	Measure three-month effectiveness of TBW techniques in severe behavioral disorders in children with PDD (pervasive developmental disorder).<br>A secondary objective is to evaluate the place of TBW in the global therapeutic strategy to offer the most seriously autistic children with autism.
Plan de l'étude	Phase II therapeutic trial
Sample size	40 subjects will be recruited (2 groups of 20 subjects).
Critères d'inclusion	Children and adolescents over 3 years of age with a diagnosis according to international criteria (ICD 10) evaluated by ADI-R of: autistic syndrome, Asperger's syndrome, or pervasive non-specific developmental disorder (PDD-NOS);<br>- presenting serious behavioral disorders (such as auto or hetero-aggressiveness, self-mutilation, severe psychomotor instability, severe and invasive stereotypies);<br>- having received a neuropediatric consultation;<br>- for epileptic patients: stable dosage of antiepileptic medication for at least 4 weeks;<br>- after informed consent of the parent (s) or legal representative.
Study duration	3 years + 2 years (MS2) + 1 years (MS 7)

V- STUDY JUSTIFICATION

1.1 Autism and Pervasive Developmental Disorders (PDD)

PDD are serious and chronic disorders that hinder the child's communication and socialization abilities and are usually accompanied by a major restriction of the child's interests. In their typical form, autistic disorders begin before the age of 3 years.

While many children with autism or PDD can benefit from formal schooling, autistic disorders are often accompanied by severe behavioral disturbances such as psychomotor instability, invasive stereotypies, hetero or self-aggression, or even sometimes very serious self-injury (sometimes going to enucleation).

Children suffering from PDD should benefit as early as possible from multidisciplinary care combining intensive care (full-time hospitalization or day hospital with or without drug treatment), educational / rehabilitative care and appropriate pedagogy in their concrete way of thinking. Early and intensive care is needed because of the importance of early childhood in establishing interpersonal relationships and body image. The dynamism of development in children and adolescents justifies the population of this study.

1.2 Psychotropic treatments

There is currently no specific medication for the core symptoms of autism such as social interaction difficulties, communication and language disorders or restriction of interests. In contrast, treatments for some medically available symptoms (such as behavioral disorders) have been extensively studied, although rarely in double-blind, placebo-controlled trials (Barnard et al., 2003, Buitelaar & Willemsen-Swinkels 2000; Palermo & Curatolo 2004, McDougle et al 2003, Posey & McDougle 2001, Tsai 1999).

The only drug to benefit from an approval of the French Medical Agency for Behavioral Disorders in children with autism older than 5 years is Risperidone (Risperdal®). Risperidone is among the most studied and prescribed drugs in children and adolescents with pervasive developmental disorders. The efficacy of risperidone is demonstrated in severe behavioral disorders associated with autistic syndromes in children and adolescents such as psychomotor instability, stereotypy, aggression and self-mutilation (Aman et al 2005, Aman 2005, McDougle et al 2005, McDougle et al 1998, Posey & McDougle 2001, RUPP 2005, RUPP 2002, Scahill et al 2001, Shea et al 2004, Troost et al 2005, Williams et al 2006).

1.3 Therapeutic body wrap (TBW)

For several years, the TBW technique (or packing or wet wrapping sessions initially cold) is proposed for the treatment of children and adolescents with autism most seriously ill, or who present serious behavioral disorders (Delion et al., 1998). Given the positive clinical results obtained, it seems important today to make a systematic study that will validate their relevance. No scientific data are available at this time and the validity of this method of care should be scientifically established. Comparable research cannot be performed on adults because of the responsiveness to management related to significant plasticity in children and adolescents in development.

In spite of the passionate national polemic about packing (see the very objective and nuanced synthetic echoes published in the Lancet [Spinney, 2007]), the reviewers of the National Clinical

Research Program did not raise any difficulty in its realization from an ethical point of view. The clinical results suggest a very favorable benefit / risk ratio.

The TBW technique is based on fast thermal skin warming (of the order of 3 to 5 minutes according to skin thermometer checks) obtained by wrapping the body of the child in cold wet towels. These tissues with a temperature close to 10 ° early in the session warm up very quickly to approach the temperature of the body at the end of the session. Only the surface temperature is temporarily decreased, the patient is never placed in hypothermia. This technique favors a preferential use of thermosensitivity under its two pathways (thermo-nociceptive when the temperature is below 10 ° and thermoceptive above). The wrap sessions take place once or several times a week depending on the patient's state of health. The TBWs help the children to strengthen their awareness of the limits of their body. The purpose of the TBW is to help the patient to find a body image by focusing on his sensory and emotional experiences. Much more than a behavioral and only bodily method, the TBW allows a sense of self which allows the child to establish a relation with the caregivers who accompany the child attentively during the sessions.
The TBWs are part of an individualized care project in agreement with the parents. They never happen when they are rejected by the child. This care is part of a multidisciplinary care that combines care, education and pedagogy.
Some results (Tordjman & Charras, 2007) suggest that the observation of an apparent decrease in pain responsiveness in childhood autism is not an endogenous mechanism of analgesia, but rather a different mode of expression of pain, related to disorders in symbolizing, disorders of both verbal and nonverbal communication and other cognitive disorders such as disorders of the learning and the image of the body, problem of sensory and emotional representations, difficulties in establishing cause-and-effect relationships. A certain relationship seems to exist between the improvement of pain reactivity behaviors and the progression of body construction as evidenced by the grid of G. Haag et al. (2005).

Tordjman and Charras hypothesised that some autistic patients use self-mutilation to cause a painful sensation, knowing that self-mutilation, according to the clinical observations of caregivers and parents, appears mainly when patients are stressed. It is possible that this painful sensation would allow the patients to focus completely on themselves, that is to say to be able to reach a certain autistic withdrawal, and to cut themselves off from an anxiety environment as if this painful sensation had a hypnotic effect.
According to these authors, TBW thanks to the initial cold sensation, mobilizes the thermoalgic sensitivity via the spino-thalamic beam, and bypasses the painful sensation on which the child might have focused. It is therefore through TBW to replace a sensation which the patient has become extremely dependent by another type of stimulation mobilizing the same neuro-physiological circuit.
This "substitute" can enable him to get out of his dependence on self-mutilation and the painful sensations associated with it. According to Tordjman and Charras, it is possible that the mobilization of the circuits caused by the TBW is effective in the long term against the behaviors of self-mutilation. In other words, if the child with autism, focused on the painful sensation he causes and controls from his self-injury, now focuses his attention on the cold generated by the TBW, it is possible that we can begin a work of quitting against his self-aggressive behaviors and sensations.

Hypothesis

TBW can legitimately be offered to the most seriously ill children with PDD. TBW is offered independently of psychotropic prescriptions.

VI – STUDY AIMS

A) Primary Objective

The main objective is to measure the effectiveness at 3 months of TBW in severe behavior disorders of children with PDD.

B) Secondary Objective

A secondary objective is to evaluate the place of TBW in the global therapeutic strategy to propose to the most seriously disabled children with autism.

Another objective is to evaluate the possible different importance of any psychotropic prescriptions in each group (wet or dry TBW).

C) Primary variable

Decrease in the intensity of behavioral disturbances measured by the Aberrant Behavior Checklist (ABC) Irritability sub-score at week 12 (primary endpoint).

D) Secondary variables

- CARS between randomization and 3-month evaluation.
- Difference in total score and sub-scores of hyperactivity, lethargy, and stereotypy of the ABC scale between randomization and 3-month evaluation.

VII – STUDY DESCRIPTION

A) Methodology

1) Type of trial

Phase II trial.

2) Study design

Randomized control trial with parallel groups. Blind assessment.

B) General study organization:

1) Study centers

The search is carried out: (see appendix)

- in the university departments of child psychiatry in Lille (Pr Pierre Delion), Caen (Pr Jean-Marc Baleyte) and Amiens (Christian Mille Pr).
- in non universitary child psychiatry units in the Nord-Pas-de-Calais region, who usually practice TBW
- in some medico-social structures (Medical-Educational Institutes) which usually practice TBW

The addition of centers will be subject to amendments.

- Centre de Santé Mentale angevin CESAM (49) (Dr Charlery Martine)
- Centre hospitalier de Brive (19) (Dr Vaillant Anne Marie)
- Centre hospitalier La Pitié (75) (Dr Xavier Jean)
- Centre de Soins Médico-Psychologique pour enfants
- Service de psychiatrie infanto-juvénile du secteur 49i03 au Centre Hospitalier de Cholet(49) (Dr Stéphanie Dauver)
- Hôpital Maison Blanche - Secteur Buttes Chaumont (75) ; Hôpital de jour « La pomme » (paris 18ème) : Dr Fabienne Roos-Weil
- CH de Lens (62) – Dr Batardière Nathalie
- Hôpital de jour Enfants de Gennevilliers (92) : Dr Louzoune Claude.
- Centre hospitalier de Montfavet (84) : Dr Bonnauron Christine.
- IME la pépinière à Loos (59) : Dr Catot-Larue Claude
- IMA de Montigny en Ostrovent (59) : Dr Lepintre Claude
- EPSM val de lys/artois à St Venant (62) : Dr Gaudry Caroline.
- Centre Hospitalier d’Arras (62)- service pédopsychiatrie 62i07 : Dr Ardouin Geoffrey
- Centre Hospitalier George Mazurelle (85 La Roche sur Yon) : Dr Benbouhou Florence
- Centre Hospitalier de Marne la Vallée - Hôpital de jour l’Olivier (77 Jossigny/ Lagny) : Dr Duguet Marion (MS7)

2) Accueil des sujets et déroulement de l'étude

1. Recruitment, parents information, reflection period

Patients are screened and recruited by medical discussion in the ambulatory units and hospital departments of the centers included in the study. The possible patient selection is discussed when TBW support is offered to a child or adolescent meeting the inclusion criteria of the study.

The local investigator proposes to the parents the participation in the study, provides them with the information and consent form and invites them to watch the "Cocon de Soi" DVD produced by the parents of an autistic child who has benefited from TBW. Parents have a reflection period of one week (or more if they wish).

2. **Pre-inclusion visit**

The local investigator will personally examine the patient and proceed with pre-inclusion in the study. The local investigative doctor informs parents about the progress of the study and has them sign the information and consent form.
The doctor organizes the first psychological evaluation of the child.

3. **Inclusion visit, Randomisation, 1st psychological assessment**

The doctor responsible for the patient's care realizes the inclusion in the study with the participation of the dedicated team of Lille University Hospital.

An anonymization number is assigned by telephone call from the local investigator to Lille University Hospital. Randomization definitively determines the mode of management: 1) Arms with wet TBWs; 2) Arms with dry TBW (blankets without wet cloth).

The psychologist carries out with the child, the team and the parents the first psychological assessment (ABC, SIBS, CGI, observation notebook) before any envelopment.

4**. Wet and dry TBW**

Wet or dry wraps start according to the modalities provided by the protocol (at the rate of two sessions per week). The wraps are performed by two members of the health care team of the facility where the patient is usually in care, or by that of the hospital unit (full time or day) where the child is hospitalized. Depending on the needs, this team may be assisted or assisted by nurses recruited by PHRC funding. The staff will be trained in the TBW technique by the team of the department of child and adolescent psychiatry of Professor Pierre Delion, University Hospital of Lille. Health and medico-social collaborations are possible.

The psychologist performs blind assessment (i.e. ignoring the mode of care) on the place of care, with the child, the team and parents (ABC, CARS, SIBS, CGI, observation booklet) at the very beginning of inclusion (before the start of wraps), then after 1, 2 and 3 months.

5. **Follow-up of the study at 1, 2 and 3 months**

At each monthly follow-up visits, the psychologist performs the psychological assessments. The local investigator ensures the smooth running of the study and reports monthly on a logbook

(treatment schedule) the clinical developments that will be controlled for data accuracy by the Research Clinical Assistant on the observation book.

At the end of the 3-month study, the observation book is sent to the principal investigator at Lille University Hospital. The data will then be entered by a Clinical Research Associate funded by the PHRC.

6. **End of study**.

After the 3-month treatment study, the child leaves the study protocol, the continuation of care being under the responsibility of the doctor locally responsible for its usual care.

a. In the case where the dry TBW prove ineffective, their stop is possible (after the 3 official months). In this case, the realization of wet TBW and initially cold is possible outside the framework of the study by decision of the doctor responsible for the care of the child.

b. In the case where wet or dry TBW have been effective, their continuation is of course always possible according to the opinion of the doctor responsible.

Definitions

Wet TBW: wraps in wet bath towels and initially cold (about 10-15 ° C). The towels are covered with blankets to facilitate the warmth of the child (in 3 to 5 minutes in practice). The sessions last about 45 minutes. The rhythm is two sessions per week during the 3 months of the study. The number of sessions (wet or dry) per week must be respected afterwards. The subject is constantly accompanied by the trained people who perform the wrap. At the end of the session, the subject is rubbed and accompanied to the place of life where a snack can be proposed in a friendly atmosphere.

Dry TBW: wraps in a simple dry cover in the same conditions of placement of the body, rhythm and mobilized staff as during the TBW sessions.

Remark : The wraps flexibly limit the mobility of the child. He cannot get out of the clothes, but he can move and stand up. It is not a restraint with shackles on a bed. Only the upper and lower limbs and the trunk are wrapped. The head and face are not concerned.

VIII – PARTICIPANTS' DESCRIPTION

The study addressed patients who are sick, minor and unable to give their consent (without language or too few).

A) Sample size calculation

This is a pilot study. We propose to recruit 20 subjects per group in order to estimate with sufficient precision the effect size. The effect size is equal to the difference of the averages observed between the groups, divided by the estimated standard deviation. It thus makes it possible to normalize the observed difference with respect to the standard deviation.

According to Cohen (Cohen J, Power Primer, Psychological Bulletin 112 (1): 155-159, 1992), the effect size is interpreted as follows: 0.2 corresponds to a low effect size, 0.5 to a medium effect size and 0.8 to a large effect size. The estimate of effect size for this study will be interpreted according to these rules. This study should determine if the effect size is sufficient.

B) Inclusion criteria

There is no reason to exclude children with genetic abnormalities or a particular neurological pathology. In the particular case of epilepsy, the treatment must be stable (see protocol).

The Intellectual Quotients (WISC, K-ABC) and the Development Quotients (Brunet-Lezine) and the Psycho-Educational Profile (PEP-R) do not intervene in the inclusion criteria, but their information is useful (among the children and adolescents who can be tested despite their possible impairments or serious behavioral disorders) for the description of the study population, in particular in order to evaluate the eventual different effectiveness of TBW according to IQ.

A neuropediatric assessment is part of the usual clinical evaluation of children and adolescents with autism. Its realization can take place during or after the TBW. Complementary examinations (EEG, CT scan, MRI, karyotype, X-fragile, search for the MECP-2 gene, etc.) are prescribed outside the scope of the study.

Criteria :

- Children and adolescents over 3 years of age presenting according to the international criteria (ICD 10) evaluated by ADI-R: an autistic syndrome, Asperger's syndrome, or an invasive non-specific development;
- Presenting serious behavioral disorders (such as auto- or hetero-aggression, self-mutilation, severe psychomotor instability, severe and invasive stereotypies) objectified by the scores: sub-score Irritability of the ABC scale> 18, CGI> 4;
- Having received a neuropediatric consultation (which will determine the opportunity to carry out additional examinations);
- For epileptic patients: dosage of stable antiepileptic treatment for at least 4 weeks;
- After informed consent of the parent (s) or legal representative.

C) Non-inclusion criteria

- Subjects with Rett syndrome or childhood disintegrative disorder;
- subjects with a history of neuroleptic malignant syndrome;
- lack of parental consent after information;

D) Expected benefits for the participant

Decrease in severe behavioral disorders, stereotypies, hyperactivity, insomnia, self- or hetero-aggressive behavior.

Better awareness of body image.

Improvement of the relational contact with others, improvement of the quality of life (decrease of the frequency and the times of contention)

E) Criteria for participant's withdrawal from the study

Each participant will be able to leave the study in case of impossibility of TBW realization, of manifest refusal on his part, or of refusal of one of his parents. The data already collected are taken into account in the final description of the study.

Each participant may leave the study by decision of the competent administrative authority, the sponsor and the coordinating investigator but also by decision of a co-investigator or by decision of the interested party himself in accordance with the regulations and as it is mentioned in the consent.

IX – TRAITMENTS

Inclusion in the study is not dependent to any prescription.

Concomitant treatments before and/or during the trial are

- authorized: all treatments. Any modification of the drug treatment (addition or withdrawal of a drug, increase or decrease in dosage) during the experiment should be justified.
- prohibited: any experimental treatment.

Prescription is performed under the responsibility of the doctor responsible for the overall care of the child, in no case it can be motivated by inclusion in the protocol.

X – BIOLOGY

No additional complementary work-up is required for the study: the clinical and laboratory evaluations conducted during this study will be consistent with the recommendations of assessments during the management of a child or adolescent with an PDD, and the medical supervision recommendations for antipsychotic treatment if needed, including:

A measurement of weight and height at the beginning and the end of the study is important because the body mass index (BMI=P/T^2) to be adjusted to the sex and age of the child. This is a convenient and reliable way of monitoring possible weight gain, especially in cases where a prescription for psychotropic drugs is needed..

Other measures are helpful: Blood Pressure, Waist Round, Hip Round, Fasting Blood Glucose, Total Cholesterol and Triglycerides, but no blood test is required for inclusion in the study.

Likewise, no ECG recording is needed for inclusion in the study.

XI – SERIOUS ADVERSE EVENTS

A) Definitions

Adverse event

Any adverse event occurring in a person, who is amenable to biomedical research, whether or not that event is related to the research or product to which the research relates, is considered an adverse event.

Serious adverse event

The following are considered serious adverse events: deaths, and all events that occur during the study and that:
- are life-threatening,
- require an extension of hospitalization,
- result in irreversible lesions or sequelae,
- are considered serious by the investigator.

Unexpected adverse event

An adverse event should be considered unexpected if its nature, intensity, or evolution are not consistent with the information in the protocol.

B) Adverse events and foreseeable risks related to the protocol

The foreseeable risks related to the protocol are:
- risks related to the TBW technique:

Fall on the floor from the height of the bed where the child is lying to be wrapped. In case of difficulty, wraps can be made with the mattress on the floor.

Supervisory Committee:
It has an advisory function when the sponsor calls on him on medical points such as tolerance and adverse events. It is made up of people outside the study, including a clinician specialized in the pathology studied (Professor Claude Bursztejn, University Hospital of Strasbourg), a member of an association of parents of autistic children (Ms. Ghislaine Meillier, former President of the Association Sesame Autism North) and a pharmacologist (Prof. Régis Bordet, Institute of Predictive Medicine and Therapeutic Research, INSERM, IFR 114).

C) Declaration

The investigator must notify the sponsor, without delay from the day he becomes aware of it, of all serious adverse events.

All serious adverse events should be reported on a "Serious Adverse Event" form in the compliance record. This form must be sent to the sponsor (Vigilance Cell of the Clinical Research Federation) by fax on 03 20 44 57 11.

The follow-up of the adverse events will be ensured by the investigator until the disappearance of the symptom.

The causal relationship between the event and the products of the study can be of several kinds:
- without a causal relationship with biomedical research,

- with causal relation:
  *doubtful
  *possible
  *likely

All events deemed either by the investigator or by the sponsor as not being "unrelated to biomedical research" are suspicions of adverse events.

The sponsor is responsible for reporting to DGS and PPC the serious and unexpected adverse events attributable to the study procedures and/or medication used within a delay of 7 days maximum

XII – STATISTICAL ANALYSIS PLAN

Detailed statistical analysis plan:

The statistical analyzes will be carried out using the SAS software (version 9.3 or higher) and conducted at the methodological support platform of the Lille University Hospital under the supervision of Professor A. Duhamel. All statistical tests will be bilateral with a risk of the first species of 5%. The quantitative variables will be described by the mean and the standard deviation in case of Gaussian distribution, or by the median and the interquartile (ie 25th and 75th percentiles) in the opposite case. The normality of the distributions will be tested by a Shapiro-Wilk test and checked graphically by histograms. The qualitative variables will be described by the numbers and percentages of each category.

Sample of analysis:

Since this is a Phase II trial with the primary objective of estimating the effect of the TBW technique, all analyzes will be performed on the Per-Protocol (PP) sample. The PP population is defined by randomized patients without major deviation from the protocol. The major deviations are the following: wrongly included, untreated patients (without a wrap-up session, whatever the arm of the study), and patients for whom the main criterion is not available, including drop-out of the study. The total number of randomized patients, the number of patients included in the per-protocol analysis and the details of the major deviations will be described in the form of a flow-chart.

Group comparability:

Baseline characteristics (before treatment) will be described and compared between the two groups to ensure group comparability. Quantitative variables will be compared by a Student's t-test or a Mann-Whitney U-test if there is a deviation from normality. Qualitative variables will be compared between groups by a Chi-square test or Fisher's exact test (when Chi-square test validity conditions are not verified).

Analysis of the main criterion:

The primary endpoint (change in ABC irritability score) will be compared between the two groups by a covariance analysis adjusted for irritability score at entry (before treatment). We will estimate from this model, the difference of the means of change of the score (experimental vs control) by 95% confidence interval. The effect size associated with the use of the TBW will also be calculated (standardized mean difference, "Cohen's index"); the 95%-fold confidence interval of the effect size will be calculated by bootstrap (re-sampling). A diagnosis of validity of the model will be made (normality of the residues, influential values). In the event of a deviation from normality of residues, the relative difference in irritability score between randomization and 3 months will be calculated and compared between the two groups by a Mann-Whitney U test.

Analysis of secondary criteria:

The frequency of patients with a clear improvement in overall clinical impression will be compared between the two groups by a Chi-square test or Fisher's exact test. The effect size will be calculated by the difference in absolute and relative frequency (experimental vs control).
The same analysis strategy will be used to compare the frequency of patients with a marked improvement in overall clinical impression between the two groups.
The analysis strategy described for the primary endpoint analysis will be used to compare the

change in ABC total score, hyperactivity sub-scores, lethargy, and ABC scale and CARS score stereotypy, between the two groups.

Exploratory analyses*:*

A first exploratory analysis will be carried out on the set of repeated measures of the total score and ABC sub-scores (M0, M1, M2, M3). The repeated measurements will be compared between the two groups by a mixed linear model. This model is an extension of the classical analysis of variance which makes it possible to take into account the correlation between two successive measurements of a patient. The choice of the covariance structure will be based on the AIC criterion.
A second exploratory analysis will be carried out to study the link between age and the main criterion on the one hand, and between the number of TBW sessions and the main criterion on the other hand. The Spearman correlation coefficient will be calculated in each group separately.
A third exploratory analysis will be performed by adjusting the comparison of the primary endpoint to the age of patients, including age in the covariance analysis model. If the main analysis is done by the Mann-Whitney U-test, a data rank covariance model (non-parametric) will be used.

XIII – STUDY DURATION

Expected duration for recruitment: 3 years + 2 years + 1 year
Duration of the study, from the first inclusion to the last patient's last visit: 6 years and 3 months
Duration of participation planned for a given subject: (preparation, treatment: between 1 and 3 months, follow-up: 3 months)
The trial will end when more than 40 subjects have been included, but should not exceed 72 months in total (until December 2014).

The sponsor reserves the right to discontinue the trial due to lack of inclusions.

There is no reason to prohibit the inclusion of children and adolescents included in this protocol in protocols other than those aimed at assessing the impact of the care of children and adolescents suffering from PDD with serious behavioral disorders.

The study may be stopped by decision of the competent authority, the sponsor or the coordinating investigator.

XIV - RESPONSIBILITIES (LEGAL AND PRACTICAL)

A) Necessary means

1) *Necessary staff*

TBW sessions will be carried out by at least 2 staff (state nurses or psychomotor nurses or educators) who will accompany the subject during the sessions.

Psychological assessments will be done at inclusion and then at M1, M2, M3 and M4 by a clinical psychologist.

The data will be entered by a clinical researcher (3 ManMonths) per year for all assessment centers).

2*) Financial means*

Funding through PHRC 2007/1918 allows the hiring and relocation of 3 FDI full-time (6 FDI half-time), three half-time psychologist and one half-time ARC.

B) Type of participation expected for the different actors

	Investigateur principal	*Investigateurs*	*Promoteur (DRC)*	*Autre, préciser :service de soins*	*Charge de travail (en nombre d'heures)*
**Accompagnement méthodologique (protocole CRF)**	x		x		
**CV datés et signés**	x	x			
**Soumission CPP et Ministère**			x		
**Assurance**			x		
**Déclarations administratives**			x		
**Convention financière**			x		
**Elaboration du CRF**	x		x		
**Mise en place**	x		x		
**Stockage des traitements**				x	
**Recrutement des patients**	x	x			
**Recueil du consentement/Information du patient**	x	x			
**Randomisation**	x	x	x		
**EIG**	x		x		
**Pharmacovigilance**			x		
**Monitoring/Suivi de l'état d'avancement**			x		
**Recueil de données/remplissage des CRF**	x	x		x	
**Stockage prélèvements/Traçabilité**	x	x		x	
**Amendements**			x		
**Fermeture du (ou des) centre(s)**	x		x		
**Analyse des données**	x			x	
**Clôture**			x		
**Rapport final**	x				
**Archivage**			x		

C) Submission to the CPP for opinion and to the DGS for authorization

The sponsor has submitted the protocol for opinion to the CPP Nord Ouest and for authorization to the DGS in accordance with the law n ° 2004-806 of August 9, 2004 relating to the public health policy and its decree of application n ° 2006-477 of the April 26, 2006 and the related orders and decisions.

D) Amendments to the protocol

There will be no alteration or change to this protocol without the agreement of all investigators and the sponsor.

In accordance with the provisions of article R 1123-35 of the public health code, the substantial modifications are those that have a significant impact on any aspect of the research, in particular on the following criteria:
• the protection of persons, including with regard to their safety
• the validity conditions of the search
• where appropriate, the quality and safety of the products tested (experimental drugs and any other products used in the research)
• the interpretation of scientific documents that support the research process
• how to conduct research

Only substantial changes are subject to authorization and/or opinion of the DGS and the CPP. Non-substantial changes are forwarded to the CPP for information.

E) Subject Information, Written and Informed Consent

Complete and fair information (in terms easily understood by the subject) must be provided and a newsletter (see appendix) must be provided to the subject by the investigator or his / her appointed representative.

It specifies the objectives, the methods and the duration of the participation (for a given subject), the main constraints of the protocol, the possible risks incurred and the security measures taken. It recalls that the subject may refuse, and at any time and without personal prejudice, withdraw his consent and that he may be informed of the overall results of the study when it is completed.

An informed consent form (see appendix) will also be drafted.

The subject's adherence to the search will not be sought. Information will be produced using, for example, a doll wrapped in strips of cloth and watching the DVD "A Cocoon of Self" made by the parents of an autistic child who has benefited from TBW.

The form of collection of consent is signed, as the case may be:
- by the two holders of parental authority (parents),
or
- by the legal representative of the minor, or the family council or the guardianship judge, if the minor is under guardianship.

The newsletter and the informed consent form are signed by the child's parents or any other person who authorizes participation in the research (e.g. legal representative, guardianship judge), as well as by the declared investigator.

A copy of these documents will be given to the persons authorizing the child's participation in the research; another will be kept by the investigator.

Only a declared investigator can sign these documents.

F) Protocol observation books

A protocol observation booklet must be completed for each patient included in the study. This notebook will be anonymised to ensure the confidentiality of the data.

During the study, the notebooks will be kept in the investigator's office and filled in by the investigator.

At the end of the study once the closure is done, the observation books will be grouped together and kept at Lille University Hospital Center.

G) Quality assurance

The study will be followed by a Clinical Research Associate who, at regular intervals, will ensure that the study is conducted in accordance with the protocol, Good Clinical Practice and applicable regulations.

He will be responsible for verifying the notebooks to ensure that they are completely and clearly completed and that the data are consistent with the source documents.
The investigator assures the sponsor of his cooperation in this verification.
In addition, the investigator and the sponsor may be subject to inspection by the competent authorities.

A monitoring of the study will be carried out according to the model of the risk B i.e. predictable risk close to that of the usual care (relates to the clinical trials of therapeutic researches on the use of standardized techniques, but whose efficiency does not is not demonstrated).

The monitoring will focus on:
- the existence of patients included
- informed consents to be signed
- the eligibility criteria
- the main criterion of judgment
- adverse events and Serious AE
- the management of the products under study.

XV - CONFIDENTIALITY

The data will be anonymous and listed according to a region, center and patient number.

The investigator will retain for his use a list of subjects associating identities, addresses, telephone numbers and file numbers, with the numbers, initials or codes under which they appear on the documents relating to the study protocol. This list will be archived with the documents relating to the study protocol.

The data and samples will be treated confidentially (medical secret) and it will not be possible for the sponsor to know or guess the identity of a subject. The data processing will be carried out under the conditions of confidentiality defined by the relative to the data processing, the files and the liberties, modified by the law N ° 2004-801 of August 6, 2004 and the regulatory texts taken for its application.

XVI – INSURANCE

The CHRU as Sponsor will have taken out an insurance contract "Membership in the Promoting Contract of Biomedical Research" (SHAM, Lyon France), guaranteeing, in its conditions and limits, the legal civil liability that may fall on the investigator and his collaborators because of the damage caused to the subjects and resulting from the present research carried out in accordance with the instructions of the protocol and in compliance with the regulations and professional practices in force.

XVII- FINANCIAL CONVENTIONS

The budget of the study is under the responsibility of the promoter who must ensure that it is sufficient for the proper conduct of the study.
The project received funding under the PHRC 2007.

XVIII – FINNAL REPORT AND PUBLICATIONS

The results of the study will be proposed for publication at the end of the study in international journals of psychiatry. Dr. Jean-Louis Goeb and Pierre Delion will be co-authors, after analyzing the data. Mention will be made in these publications of the role of the sponsor: University Hospital of Lille.

The final report of the study will be written by Dr. Jean-Louis Goëb, and Professor Pierre Delion.

If any patents, directly resulting from this study, were filed, this would be done after consultation with the sponsor of the study, and the scientific and financial benefits would be distributed after prior agreement between the various partners (CHRU Lille, INSERM, University of Lille 2 and Pasteur Institute of Lille) in proportion to their current work and their financial participation in patent filings.

XIX – INVESTIGATOR’S RESPONSABILITIES

Each investigator undertakes to scrupulously respect the protocol and the rules of Good Clinical Practice (GCP).

He undertakes to accept the controls of an ARC and to provide access to the source data (medical files, computer files, study documents ...).
During these site visits in accordance with GCP, the following elements are reviewed:
- respect for the protocol and related procedures
- quality assurance of the data collected in the report: accuracy, missing data, data consistency, control of source documents.

The investigator undertakes to keep source data as well as the administrative documents related to the protocol and not to include a participant before receiving the official notice of the CPP and sending the declaration to the authorities, to respect the protocol, to conduct the study according to the moral, regulatory, ethical and scientific principles that govern clinical research. He also undertakes to collect the informed and written consent of each participant and to report any serious adverse event.

He enters the search into a European database.

Duration of the archiving of study data and consents: 15 years.

XX - ANNEXES

- Newsletter and informed consent form (see appendices 1 and 2)

- Budget of the study and financial agreements (cf appendix 3)

- Abstract CV of the investigators with their publications (see appendix 4).

REFERENCES BIBLIOGRAPHIQUES

Aman MG, Arnold E, McDougle CJ, et al. Acute and long-term safety and tolerability of risperidone in children with autism. J Child Adolesc Psychopharmacology 2005 ; 15 : 869-884.

Aman MG, Singh NN, Stewart AW, et al. The Aberrant Behavior Checklist : A behavior rating scale for the assessment of treatment effects. Am J Ment Deficiency 1985 ; 89 : 485-491.

Aman MG. Treatment planning for patients with autism spectrum disorders. J Clin Psychiatry 2005; 66 Suppl 10 : 38-45.

Arnold LE, Aman Mg, Martin A, et al. Assessment in multisite randomised clinical trials of patients with autistic disorders: The autism RUPP Network. J Autism Dev Disord 2000 ;30 : 99-111.

Barnard L, Young AH, Pearson J, et al. A systematic review of the use of atypical antipsychotics in autism. J Psychopharmacol 2003 ;16 : 93-101.

Buitelaar JK, Willemsen-Swinkels SHN. Medication treatment in subjects with autistic spectrum disorders. Eur Child adolesc Psychiatry 2000; 9 : I/85-I/97.

Carter AS, Volkmar FR, Sparrow SS, et al. The Vineland Adaptative Behavior Scale : Supplementary norms of individuals with autism. J Autism Dev Disord 1998; 28: 287-302.

Correll CU, Carlson HE. Endocrine and metabolic Adverse effects of psychotropic medications in children and adolescents. J Am Acad Child Adolesc Psychiatry 2006; 45: 771-91.

Correll CU, Penzer JB, Parikh UH, et al. Recognizing and monitoring adverse events of second-generation antipsychotics in children and adolescents. Clin Adolesc Psychiatric N Am 2006;15: 177-206.

Correll CU. Metabolic Side effects of second-generation antipsychotics in children and adolescents : A different story ? J Clin Psychiatry 2005 ; 66 : 1331-2.

Delion P. Le packing avec les enfants autistes et psychotiques. 1998 : Erès ; Toulouse.

Diagnostic and statistical manual of mental disorders, 4th ed. DSM-IV. Washington, DC : American Psychiatric Association ; 1994.

Fenton WS, Chavez MR. Medication-induced weight gain and dyslipidemia in patients with schizophrenia. Am J Psychiatry 2006 ; 163 : 1697-1704.

Haag G, Tordjman S, et al. Grille de repérage clinique des étapes évolutives de l'autisme infantile traité. Psychiatrie de l'enfant 1995 ; 38 : 495-527.

Lord C, Pickles A, McLennan J, et al. Diagnosing autism : analyses of data from the Autism Diagnostic Interview. J Autism Dev Disord 1997 ; 27 : 501-17.

Lord C, Rutter M, Le Couteur AM. Autisme Diagnostic Interview-Revised : A revised version of a diagnostic interview for caregivers of individuals with possible pervasive developmental disorders. J Autism Dev Disord 1994 ; 24 : 659-85.

McDougle CJ, Holmes JP, Carlson DC, et al. A double-blind, placebo-controlled study in adults with autistic disorder and other pervasive developmental disorders. Arch Gen Psychiatry 1998 ; 55 :633-41.

McDougle CJ, Scahill L, Aman MG, et al. Risperidone for the core symptom domains of autism : Results from the study by the Autism Network of the Research Units on Pediatric Psychopharmacology. Am J Psychiatry 2005 ; 162 : 1142-8.

McDougle CJ, Stigler KA, Posey DJ. Treatment of aggression in children and adolescents with autism and conduct disorders. J Clin Psychiatry 2003 ; 64 suppl 4 : 16-25.

Palermo MT, Curatolo P. Pharmacologic treatment of autism. J clin Neurol 2004 ; 19 : 155-164.

Posey DJ, McDougle CJ. Pharmacotherapeutic management of autism. Exp Opin Pharmacother. 2001 ; 2 : 587-600.

Psychopharmacology Bulletin. Special features : Rating scales and assessment instruments for use in pediatric psychopharmacology research. Psychopharm Bull 1985 ; 21 : 713-111.

Research Units on Pediatric Psychopharmacology Autism Network. Risperidone treatment of autistic disorder : Longer-term benefits and blinded discontinuation after 6 months. Am J Psychiatry 2005 ; 162 : 1361-9.

Research Units on Pediatric Psychopharmacology Autism Network. Risperidone in children with autism and serious behavioral problems. N Engl J Med 2002 ; 347 : 314-21.

Scahill L, McCraken J, McDougle CJ, et al. Methodological issues in designing a multisite trial of risperidone in children and adolescents with autism. J Child Adolesc Psychopharmacology 2001 ; 11 : 377-88.

Spinney L. Therapy for autistic children causes outcry in France. Lancet 2007 ;370 :645-646, 25 août 2007.

Schopler E, Reichler RJ, De Vellis RF, et al. Toward objective classification of childhood autism : Childhood Autism Rating Scale (CARS). J Autism Dev Disord 1980 ; 10 :91-103.

Shea S, Turgay A, Caroll A, et al. Risperidone in the treatment of disruptive behavioral symptoms in children with autistic and other pervasive developmental disorders. Pediatrics 2004 ; 114 : 634-41.

Tordjman S, Charras K. Intérêts d'une meilleure compréhension de l'apparente insensibilité à la douleur et des automutilations dans l'autisme : vers de nouvelles perspectives thérapeutiques. In: Delion P (Ed) La pratique du packing avec les enfants autistes et psychotiques en pédopsychiatrie. Ramonville: Eres; 2007. Pp 49-61.

Troost PW, Lahuis BE, Steenhuis MP, et al. Long-term effects of risperidone in children with autism spectrum disorders : A placebo Discontinuation study. J Am Acad Child Adolesc Psychiatry 2005 ; 44 : 1137-44.

Tsai LY. Psychopharmacology in autism. Psychosomatic Medicine 1999 ; 61 : 651-665.

Vieweg WVR, Sood AB, Pandurangi A, et al. Newer antipsychotic drugs and obesity in children and adolescents. How should we assess drug-associated weight gain ? Acta Psychiatr Scand 2005 111 :177-84.

William SK, Scahill L, Vitiello B, et al. Risperidone and adaptative behavior in children with autism. J Am Acad Child Adolesc Psychiatry 2006 ; 45 : 431-9.
